# Applied Machine Learning Techniques to Diagnose Voice-Affecting Conditions and Disorders: Systematic Literature Review

**DOI:** 10.2196/46105

**Published:** 2023-07-19

**Authors:** Alper Idrisoglu, Ana Luiza Dallora, Peter Anderberg, Johan Sanmartin Berglund

**Affiliations:** 1 Department of Health Blekinge Institute of Technology Karslkrona Sweden; 2 School of Health Sciences University of Skövde Skövde Sweden

**Keywords:** diagnosis, digital biomarkers, machine learning, monitoring, voice-affecting disorder, voice features

## Abstract

**Background:**

Normal voice production depends on the synchronized cooperation of multiple physiological systems, which makes the voice sensitive to changes. Any systematic, neurological, and aerodigestive distortion is prone to affect voice production through reduced cognitive, pulmonary, and muscular functionality. This sensitivity inspired using voice as a biomarker to examine disorders that affect the voice. Technological improvements and emerging machine learning (ML) technologies have enabled possibilities of extracting digital vocal features from the voice for automated diagnosis and monitoring systems.

**Objective:**

This study aims to summarize a comprehensive view of research on voice-affecting disorders that uses ML techniques for diagnosis and monitoring through voice samples where systematic conditions, nonlaryngeal aerodigestive disorders, and neurological disorders are specifically of interest.

**Methods:**

This systematic literature review (SLR) investigated the state of the art of voice-based diagnostic and monitoring systems with ML technologies, targeting voice-affecting disorders without direct relation to the voice box from the point of view of applied health technology. Through a comprehensive search string, studies published from 2012 to 2022 from the databases Scopus, PubMed, and Web of Science were scanned and collected for assessment. To minimize bias, retrieval of the relevant references in other studies in the field was ensured, and 2 authors assessed the collected studies. Low-quality studies were removed through a quality assessment and relevant data were extracted through summary tables for analysis. The articles were checked for similarities between author groups to prevent cumulative redundancy bias during the screening process, where only 1 article was included from the same author group.

**Results:**

In the analysis of the 145 included studies, support vector machines were the most utilized ML technique (51/145, 35.2%), with the most studied disease being Parkinson disease (PD; reported in 87/145, 60%, studies). After 2017, 16 additional voice-affecting disorders were examined, in contrast to the 3 investigated previously. Furthermore, an upsurge in the use of artificial neural network–based architectures was observed after 2017. Almost half of the included studies were published in last 2 years (2021 and 2022). A broad interest from many countries was observed. Notably, nearly one-half (n=75) of the studies relied on 10 distinct data sets, and 11/145 (7.6%) used demographic data as an input for ML models.

**Conclusions:**

This SLR revealed considerable interest across multiple countries in using ML techniques for diagnosing and monitoring voice-affecting disorders, with PD being the most studied disorder. However, the review identified several gaps, including limited and unbalanced data set usage in studies, and a focus on diagnostic test rather than disorder-specific monitoring. Despite the limitations of being constrained by only peer-reviewed publications written in English, the SLR provides valuable insights into the current state of research on ML-based voice-affecting disorder diagnosis and monitoring and highlighting areas to address in future research.

## Introduction

### Voice-Affecting Disorders

Voice and speech production relies on complex and multiorgan cooperation. The basic mechanics of speech and voice creation is that the airflow obtained by releasing the pressure in the lungs reaches the vocal folds in the larynx and vibrates the vocal cords that result in voice, and by articulating this voice speech is created [1]. The harmony between complex biological systems involved in voice and speech production leads to normal voice formation. However, at the same time, the functional dependency of several biological structures makes the voice vulnerable to being affected by diverse conditions, which may result in a pathological or disordered voice named hoarseness (ie, dysphonia).

The anomalies and the absence of vocal quality in relation to pitch, height, resonance, and duration, which are unexpected for individuals, regardless of their gender and age are characteristics of a disordered voice [2-5]. There is no globally accepted nomenclature for voice disorders. In general, structural, inflammatory, traumatic, systemic, aerodigestive, psychiatric and psychological, neurological, and functional voice disorders are substantial categories of voice problems [6]. This can be diagnosed by a health care specialist through several examinations and tests. The current approach for the diagnosis of voice disorders relies on clinical examinations consisting of interviews, perceptual voice evaluation, patient-reported outcome measures, laryngoscopy, aerodynamic assessment, voice profile, acoustic analysis, and laryngeal electromyography [7], which is time-consuming for both the patients and the clinicians and generates a high economic burden on society [8]. The appraisal based on the assessment of biomarkers gathered through clinical examinations is a crucial step that leads to a diagnosis. Here, it is necessary to point out that the clinicians do not diagnose dysphonia; instead, the target of the clinical examination is to identify the condition that leads to dysphonia, which will be addressed in this study as a voice-affecting disorder.

### Voice as a Digital Biomarker

Measurable, reliable, and repeatable assets that can be correlated with a clinical outcome are defined as biomarkers. The criteria and context of use describe the category of biomarkers such as diagnostic, monitoring, pharmacodynamic/response, predictive, prognostic, and digital biomarkers [9]. Traditional biological markers (ie, biomarkers) are used to detect molecular changes associated with diseases and have been integrated with clinical practices for decades [10]. Digital biomarkers refer to measures or features collected by digital devices [11,12] and are a developing landscape that shares the same objectives as traditional biomarkers in answering health-related questions [13].

As aforementioned, voice and speech can be influenced by several conditions and disorders, which contribute to decreased quality of life. Nevertheless, being so sensitive could open possibilities for earlier diagnosis of disorders that affect the voice through the use of voice as a biomarker [14]. As the collection of voice and speech is a noninvasive process that can be performed at a low cost [11], the voice as a digital biomarker could be a diagnostic and prognostic resource with the potential to be more economically viable, in addition to being a more ecological measure than many of the currently used clinical alternatives for the assessment of cognition and function [9,15].

### Machine Learning for the Assessment of Voice Signals

Most health problems could benefit from an early diagnosis for better treatment and management of outcomes. However, the growing pressure on health care systems, due to the increased life expectancy and an aging population, may hinder this early detection. Patients are usually referred to specialist care only when apparent signs of disease are present, and thus are at an already moderate advanced state. Fortunately, the existence of digital biomarkers (eg, voice), along with the trend of digitalization in health care, opens the possibility of using technologies such as machine learning (ML) to address these issues. Research on biomarkers extracted from voice and speech with ML techniques for diagnosing, prognosticating, and monitoring disease [14] has shown satisfactory outcomes for disorders such as dementia, depression, mild cognitive impairment (MCI), autism spectrum disorder, Alzheimer disease (AD), and PD [14,16-18].

ML techniques are becoming prevalent in health care for aiding decision-making in treatment and diagnosis [19]. These techniques involve extracting features from voice data and using an ML algorithm to classify the severity of disorders or to determine whether a voice is pathological. The 2 most commonly used ML techniques in this context are supervised and unsupervised learning. In supervised learning, an ML technique is trained using labeled data sets (training set) and its accuracy is evaluated using unlabeled data sets (validation and test sets). The labeled data contain the actual diagnostic information that allows the ML technique to compare its output and adjust its parameters for improved accuracy. There are also end-to-end algorithms (ie, deep learning), an ML subfield that uses artificial neural networks to model and solve complex problems. These networks are composed of multiple layers of interconnected nodes that enable them to learn hierarchical representations of data. An example of deep learning in action is the use of convolutional neural networks to classify images. Convolutional neural networks use multiple layers of convolution and pooling operations to extract features from the input image and then classify it into 1 of several categories. This approach has been very successful in tasks such as object recognition, image segmentation, and speech recognition [20]. Unsupervised learning involves applying clustering methods on training data without labels to group data through 1 or several clustering algorithms [21].

Prior studies provide comprehensive information on feature extraction and its application [22-25]. Encouraging results on ML classifiers with voice biomarkers bring them into the focus of researchers. In a meta-analysis on voice disorders, Syed et al [26] applied ML techniques by setting the boundaries around 3 publicly available databases, namely, Saarbrucken Voice Database, Massachusetts Eye and Ear Infirmary, and Arabic voice pathology database. The systematic literature review (SLR) presented herein includes all possible data sources. Several reviews have investigated voice-based disease diagnostics with ML algorithms separately, focusing on only PD or AD [27-29], whereas in this study, multiple voice-affecting disorders are included.

This SLR investigates state of the art of clinical applications of voice-based diagnosis that make use of ML algorithms. Adapting voice-based diagnosis and prognosis into clinical practices requires solid evidence and research to clinically validate the usability and reliability of voice biomarkers and the performance of ML classifiers [12,15]. This SLR does not consider disorders directly related to the voice production mechanisms. Examples of included and excluded conditions are PD and polyps on the vocal cords, respectively, where PD is a neurodegenerative disorder that often causes voice changes [30] that are not directly related to voice box and polyps on the vocal cords occur in the voice box (larynx) [31]. More specifically, the conditions of interest in this SLR are listed as systematic conditions affecting voice, nonlaryngeal aerodigestive disorders affecting voice, and neurological disorders affecting voice in the *Classification Manual for Voice Disorders* [6]. These have a higher chance of being related to chronic conditions, which would benefit from having a scalable and noninvasive method for screening a large population. The expected outcome is a contribution, not only by summarizing the work done in the field of applied health technology that is interested in the application of the technology and its outcomes but also by pointing out the gaps in the literature and possible future research directions that could address the problems mentioned earlier of the next generations and the health care system.

## Methods

### Overview and Purpose of This SLR

An SLR is a summary of the results from research papers focused on a common context or a question. The summary action includes the identification, collection, assessment, and synthesizing of high-quality research evidence within the scope of the research question by following a predefined protocol. The aim of an SLR is to provide perspective on recent research so that decision makers can benefit from up-to-date knowledge and address the gaps that can be used as a basis for new research. The predefined protocol describes the methodology to follow; defines the research question; and contains information about inclusion/exclusion criteria and quality assessment [30]. This section specifies the methodology applied in this SLR to answer the research question “How is the voice as a digital biomarker being used in clinical applications that employ ML techniques for diagnosing and monitoring voice-affecting disorders?” Additionally, the main question is split into the following subquestions (SQs):

- *SQ1:* What are the aims of pathologic voice evaluation?
- *SQ2:* Which ML techniques are being used for the diagnosis and monitoring of voice-affecting disorder through voice and which voice-affecting disorders are being investigated?
- *SQ3:* What are the time and geographical trends of publications in the scope of SLR?
- *SQ4:* What are the data characteristics of the sound samples for different disorders and types of studies?
- *SQ5:* Are the studies cross-sectional or longitudinal?
- *SQ6:* How is performance being evaluated in the studies?

All the information on the methodological approach that guided the execution of this SLR is based on the prespecified SLR protocol [31].

### Search Strategy

A search string was constructed by applying the *p*opulation, *i*ntervention, *c*omparison, and *o*utcome (PICO) framework [32-34]. The most used terms, suggested by authors ALD and JSB, were used to find relevant papers; the retrieved papers and their references in the field were then used to discover new adequate keywords. By adding the new keywords to the search string, a comprehensive search string was created. A customized version of the search string in Textbox 1 was used in PubMed, Scopus, and Web of Science databases to find all relevant peer-reviewed primary journal articles published between 2012 and 2022. The application of the PICO structure excludes the *comparison* due to the nature of this SLR being a characterization:

- *Population:* Disorders that affect the voice, given by the *Classification Manual for Voice Disorders* [30], referring to the systematic conditions affecting voice, nonlaryngeal aerodigestive disorders affecting voice, and neurological disorders affecting voice.
- *Intervention:* Use of ML techniques for the diagnosis or monitoring of disorders through voice samples.
- *Outcome:* Reported quantities or results such as precision and accuracy.

The search string was adapted based on the advanced search requirements of each database. The filter options were tuned to retrieve articles from January 1, 2012 to December 31, 2022. The period was chosen after consulting with experts in the medical field with regard to the development of new technologies for health care. The development of the search string was primarily based on the MeSH (Medical Subject Headings) terms, with the help of a librarian, and categories of voice disorders in the classification manual [6].

**Search string used in PubMed, Scopus, and Web of Science databases (search date: March 13, 2023).**

( ( “Voice” OR “Linguistic features” OR “acoustic parameters” OR “Vocal features” OR “Vocal” OR “Vocal Cords” OR “Vocal biomarker” OR “Voice biomarkers” OR “ Speech” OR “Vowel” OR “Sound Spectrography” OR “Cepstrum Vectors” ) AND ( “Deep phenotyping” OR “selection” OR “extrac- tion” OR “Detection” OR “Monitoring” OR “Classification” OR “Evaluation” OR “Analysis” OR “Estimation” OR “Projection” OR “Improving” OR “Investigation” OR “Prognosis” OR “Predict*” ) AND ( “Sensitivity” OR “Accuracy” OR “Specificity” OR “Performance” OR “Cross-validation” OR “precision” ) AND ( “Voice technology” OR “Machine learning” OR “Artificial Intelligence” OR “Gaussian mixture models” OR “Support vector machines” OR “Artificial neural network” OR “Data Mining” OR “Decision Support System” OR “Clinical Support System” OR “Deep Neural Network” OR “Kernel extreme learning machine” OR “Deep Learning” ) AND ( “voice disorder” OR “systemic conditions” OR “aerogestive disorders” OR “neurologic disorders” OR “central nervous system disturbance” OR “Endocrine” OR “Hypothyroidism” OR “Hyperthyroidism” OR “Sexual Hormone Imbalances” OR “Hyperpituitarism” OR “Immunologic” OR “Allergic” OR “HIV” OR “Chronic Fatigue Syndrome” OR “Systemic Lupus Erythematosus” OR “Sjogren's Syndrome” OR “Scleroderma” OR “Wegener's Disease” OR “Musculo-Skeletal Conditions Affecting Voice” OR “Overuse Injury and Repetitive Strain Injury” OR “Fibromyalgia” OR “Ehler Danlos Syndrome” OR “Dehydration” OR “Respiratory Diseases Affecting Voice” OR “Asthma” OR “Chronic Obstructive Pulmonary Disease” OR “Digastric” OR “Gastroesophageal Reflux Disease” OR “Infectious Diseases of the Aerodigestive Tract” OR “Laryngotracheobronchitis” OR “Pertussis” OR “Diphtheria” OR “Pneumonia” OR “Infectious Sinusitis” OR “Tuberculosis” OR “Upper Respiratory Infection” OR “Acute Epiglottitis” OR “Syphilis” OR “Sarcoidosis” OR “Scleroma” OR “Leprosy” OR “Actinomycosis” OR “Mycotic Infections” OR “Blastomycosis” OR “Histoplasmosis” OR “Candidiasis” OR “Coccidioidomycosis” OR “Peripheral Nervous System Pathology” OR “Superior Laryngeal Nerve Pathology” OR “Unilateral Recurrent Laryngeal Nerve Paralysis” OR “Recurrent Laryngeal Nerve Paresis” OR “Bilateral Recurrent Laryngeal Nerve Paralysis--Peripheral” OR “Myasthenia Gravis” OR “Peripheral Neuropathy” OR “Enhanced Physiologic Tremor” OR “Movement Disorders” OR “Adductor Spasmodic Dysphonia” OR “Abductor Spasmodic Dysphonia” OR “Abductor Spasmodic Dysphonia” OR “Dystonic Tremor” OR “Essential Tremor” OR “Meige's Syndrome” OR “Tardive Stereotypies” OR “Tourette's Syndrome” OR “Amyotrophic Lateral Sclerosis” OR “Wallenberg Syndrome” OR “Lateral Medullary Syndrome” OR “Infarct” OR “Parkinson Disease” OR “Multiple Systems Atrophy” OR “Shy-Drager Syndrome” OR “Striatonigral Degeneration” OR “Sporadic Olivoponto- cerebellar Atrophy” OR “Progressive Supranuclear Palsy” OR “Multiple Sclerosis” OR “Cerebellar Disorders” OR “Huntington's Chorea” OR “Bilateral Recurrent Laryngeal Nerve Paralysis--Central” OR “Myoclonus” OR “Neuromuscular” OR “cardiovascular” OR “coronary artery” OR “heart attack” OR “Voice disorders” OR “Neurological disorders” OR “multiple sclerosis” OR “Myasthenia gravis ” OR “ALS” OR “Amyotrophic lateral sclerosis” OR “Parkinson's disease” OR “Multiple sclerosis” OR “Dementia” OR “Alzheimer's disease” OR “Essential tremor” OR “Major depressive disorder” OR “pathological voice” OR “voice pathology” OR “neurodegenerative” OR “Cognitive impairment” OR “Nodule” OR “Polyp” OR “Neoplasm” OR “dysphonia” OR “Hoarseness” OR “Huntington disease” ) )

### Study Selection

The search string was used to perform an automated search on each database. The Zotero (Corporation for Digital Scholarship) bibliography software was used to collect all relevant articles from all 3 databases and to remove duplicates [35]. First, authors AI and ALD applied the inclusion and exclusion criteria in Textbox 2 to assess the titles and abstracts of the retrieved papers. The first step was to assess randomly selected 50 papers to ensure the consistency of the criteria. Then, another batch containing 50 articles was assessed. Authors AI and ALD compared the results. Upon agreeing on the consistency of the criteria, they proceeded to assess the remainder of the papers. The degree of agreement was checked statistically by comparing the results between the first and second authors with an overall agreement of 96% using the Cohen κ index. During the evaluation, the papers were categorized into 3 groups: included, excluded, and “maybe” cases that could not be assessed by the content of the title and abstract alone. At this stage, author JSB acted as the advisor and expert in the field. Furthermore, after the evaluation of all papers, the results from both authors were cross-checked, and 30 conflicts were noticed. To minimize the risk of bias, all articles marked as included, “maybe,” and conflicts were grouped for full-text reading.

All articles in the group of full-text readings underwent a quality assessment procedure to assure high-quality evidence (Textbox 3), based on guidelines proposed by Kitchenham and Charters [36]. The quality threshold was set to 11 points, which means that articles below the score of 11 points would be rejected. The threshold of 11 points was stipulated through group discussions with authors. The questionnaire was designed in 3 sections, consisting of 5 questions each, general questions, data analysis, and results. Based on the given questions, author AI performed the quality assessment by grading the studies with scores 0, 0.5, and 1 for the sections 1, 2, and 3, respectively.

**Inclusion and exclusion criteria for the assessment of the articles.**

**Inclusion criteria**

- Journal study
- Primary study written in English
- Research published not earlier than 2012
- Research that uses voice as the input data
- Research that employs at least one machine learning algorithm
- Research that aims to diagnose or monitor at least one voice-affecting disorder not related to the systematic conditions affecting voice, nonlaryngeal aerodigestive disorders affecting voice, and neurological disorders affecting voice

**Exclusion criteria**

- A nonpeer-reviewed study
- Research written in languages other than English
- Research published before 2012 or after 2022
- Research that does not use voice as a direct input, which means research employing various nonverbal forms of data input, such as written transcriptions, digital images, videos, electroencephalogram, and signals generated during vocalization
- Research that classifies voice-affecting disease without a machine learning approach
- Research that classifies voice disorders related to conditions other than systematic conditions affecting voice, nonlaryngeal aerodigestive disorders affecting voice, and neurological disorders affecting voice

**Quality assessment questionnaire.**

**General questions**

- Are the aims clearly stated?
- Is the targeted population described?
- Has it discussed the contribution of the study?
- Are gender and age considered?
- Is/are the technique(s) being implemented clearly described?

**Data analysis**

- Is the origin of data given?
- Is the type of data clearly described?
- Do the data consist of voice recordings?
- Is the data validation method given?
- Is there a discussion on whether the data size can be generalized for the targeted population?

**Result**

- Is/are the result(s) clearly discussed?
- Are all aims or questions answered?
- Was the outcome related to the target population?
- Are the limitations discussed?
- Did results compare with previous rapports?

### Data Extraction

Data extraction was carried out by author AI. Table 1 shows the list of attributes, definitions, and purpose of use for data extraction.

**Table 1:** Collected data attributes.

Attribute	Definition
ISSN	International Standard Serial Number recorded
Title	Full title of the research
Journal	Publication venue record
Authors	All authors’ names
Publication date	The publication date of the paper
Publication type	The type of publication
Origin of publication	The geographical location of the first author’s institution
Targeted disorder	Investigated disorder
Database	Source of the data
Origin of data	The geographical location of data sources
Data characteristics	Type of voice recordings
Additional data	Used additional data except for voice recordings
Data sets	The number of participants
Sample size	The number of recordings
Aim of the study	Purpose of the study
Age range	The considered age range of the participants
Gender	The number of participants (by gender)
Quantitative result(s)	Presented outcome measures
Feature sets	Excluded features from voice
The proposed features	The best feature set, if exists
Applied ML^a^ technique(s)	All applied ML techniques
Outcome evaluation	How the pathological voice is evaluated
Type of validation(s)	How the data set is divided
Type of study	If the study is longitudinal or cross-sectional
The proposed ML algorithm(s)	ML technique with the best outcome

^a^ML: machine learning.

### Data Analysis

To analyze the etiology of changes over time and capture the heterogeneity, the studies were grouped into subgroup summary tables entitled with the name of disorders (see Multimedia Appendix 1). Numerical and statistical measures were used to represent the results. No assumption was made about the missing information. Microsoft Excel was used for data analysis. All the studies that successfully adhered to the inclusion and exclusion criteria and passed the quality assessment were eligible for data analysis. The results were presented in text, summary tables, and charts under a section for each research question. The robustness of the results was checked by conducting a sensitivity analysis through observations of the effect of some randomly removed data from summary tables [37,38]. The cumulative redundancy bias was checked by observing the similarity between author groups.

## Results

### Study Selection

**Figure 1:**
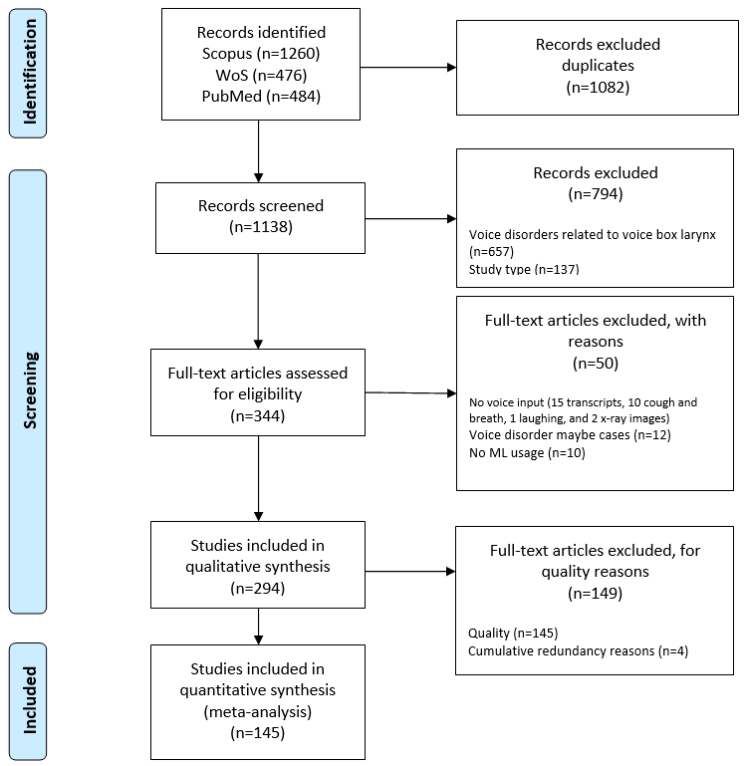
PRISMA (Preferred Reporting Items for Systematic Reviews and Meta-Analyses) flowchart. ML: machine learning; WoS: Web of Science.

The PRISMA (Preferred Reporting Items for Systematic Reviews and Meta-Analyses; also see Multimedia Appendices 2 and 3) flowchart for this study is shown in Figure 1 [39]. The automated search retrieved a total of 2220 articles from all 3 databases (Scopus, n=1260; Web of Science, n=476; and PubMed, n=484). After the removal of the duplicates, 1138 articles were assessed in the title and abstract screening. In total, 344 papers were included in the full-text reading group. During the full-text reading, 50 articles were found to be out of scope for the following reasons: related to voice box (n=12), voice was not an input (n=28; 15 transcripts, 10 coughs and breath, 1 laughing, and 2 x-ray images), and no ML technique (n=10) applied. A total of 294 articles were assessed for quality evaluation, which eliminated 145 articles and thus the final set included 149 articles that were used for data extraction.

The included papers were assessed for cumulative redundancy bias during data extraction. The assessment showed that 8 of the included papers were published by 4 different author groups, 2 papers from Tuncer et al [40,41], 2 papers from Gunduz et al [42,43], 2 papers from Lamba et al [44,45] in the PD group, and 2 papers from Tena et al [46,47] in the amyotrophic lateral sclerosis (ALS) group. To reduce the risk of bias, only 4 of those 8 papers, one with the highest accuracy from each author, were included in the synthesis [40,42,44,46]. In total, 145 studies proceeded for analysis. Sensitivity analysis did not show any effect on trend analysis, but did show a minor effect on statistical analysis. Multimedia Appendix 1 shows the list of included studies in this SLR.

### Aims of the Studies

To answer SQ1, this section sums up the aim and the assessment strategy used in studies. Many terminologies have been used to describe the aim of the studies. Generally, they can be arranged into 2 groups: diagnosis and monitoring. In the diagnosis group, 138 studies were identified. A total of 125 studies in the diagnostic group investigated ML methods to detect a pathological voice, where the participants were grouped as the healthy control (HC) group, with being “healthy” defined as people without a diagnosed disorder, and a group with known pathology [17,40,42,44,48-168]. The main idea was to deploy an ML technique for distinguishing those 2 groups from each other with high accuracy. Additionally, 13 studies [169-181] in the diagnostic group investigated ML techniques for separating several pathologies and clustered participants into several pathological groups. With the help of the ML technique, they tried to classify each group, where the primary purpose was to investigate a system that can classify multiple disorders. A total of 7 studies [182-188] were identified in the monitoring group. The pattern was trying to predict an established clinical severity assessment with the help of an ML algorithm where only participants with diagnosed disorders were involved.

### Employed ML Techniques and Voice-Affecting Disorders

Table 2 shows the results related to SQ2. A total of 19 different disorders were identified where the focus was on monitoring or diagnosis through voice or speech with ML involvement. As many as 87/145 (60%) of the studies targeted PD; 18 studies targeted dementia or AD, 8 cognitive impairment (CI)/MCI, 4 ALS, 2 cardiovascular disorders, 7 COVID-19, 2 essential tremor, 2 multiple sclerosis, 1 neurodegenerative cognitive complaint (NCC), 1 functional dysphagia/oropharyngeal dysphagia, 4 depression, 1 influenza disease, 1 neurological disease (ND), 2 stroke, 1 fatigue, 1 autism, 1 traumatic brain injury, 1 asthma, and 1 chronic obstructive pulmonary disease. NCC and ND may potentially be classified within one of either PD, AD, or CI/MCI due to their similar symptoms, but the specific underlying disorder was not provided in the studies. Therefore, these 2 disorders were grouped separately.

**Table 2:** Targeted disorders and ML^a^ techniques.

Disorder	NR^b^	ML technique (NR of usage)	References
Parkinson disease	87	SVM^c^ (34), ANN^d^ (23), RF^e^ (9), KNN^f^ (6), GB^g^ (5), GMM^h^ (2), NB^i^ (1), DT^j^ (3), SVR^k^ (2), LR^l^ (1), PA^m^ (1)	[17,40,42,44,48-102,117-135,168,170,171, 180,181,183-185,188]
Dementia, Alzheimer disease	18	SVM (8), KNN (2), LR (2), RF (3), ANN (3)	[103-110,136-139,172-176,179]
Cognitive impairment/mild cognitive impairment	8	SVM (2), LR (2), RF (2), ANN (2)	[112-116,150,177,178]
COVID-19	7	ANN (3), SVM (1), RF (1), KNN (1), GB (1)	[143-147,156,157]
Amyotrophic lateral sclerosis	4	SVM (1), RF (2), MeML^n^ (1)	[46,158-160]
Depression	4	ANN (3), SVM (1)	[140-142,161]
Cardiovascular disorders	2	KNN (2)	[111,186]
Essential tremor	2	SVM (2)	[162,163]
Multiple sclerosis	2	ANN (1), RF (1)	[149,164]
Stroke	2	ANN (2)	[148,153]
Asthma	1	RF	[182]
Autism	1	ANN	[151]
Fatigue	1	SVM	[187]
Chronic obstructive pulmonary disease	1	RF	[154]
Neurodegenerative cognitive complaint	1	SVM	[165]
Functional dysphagia, oropharyngeal dysphagia	1	RF	[166]
Influenza disease	1	KNN	[167]
Neurological disease	1	KNN	[169]
Traumatic brain injury	1	ANN	[152]

^a^ML: machine learning.

^b^NR: number of studies.

^c^SVM: support vector machine.

^d^ANN: artificial neural network.

^e^RF: random forest.

^f^KNN: K-nearest neighbor.

^g^GB: gradient boosting.

^h^GMM: Gaussian mixture model.

^i^NB: naïve Bayes.

^j^DT: decision tree.

^k^SVR: support vector regression.

^l^LR: logistic regression.

^m^PA: passive aggressive.

^n^MeML: mixed effect machine learning.

**Figure 2:**
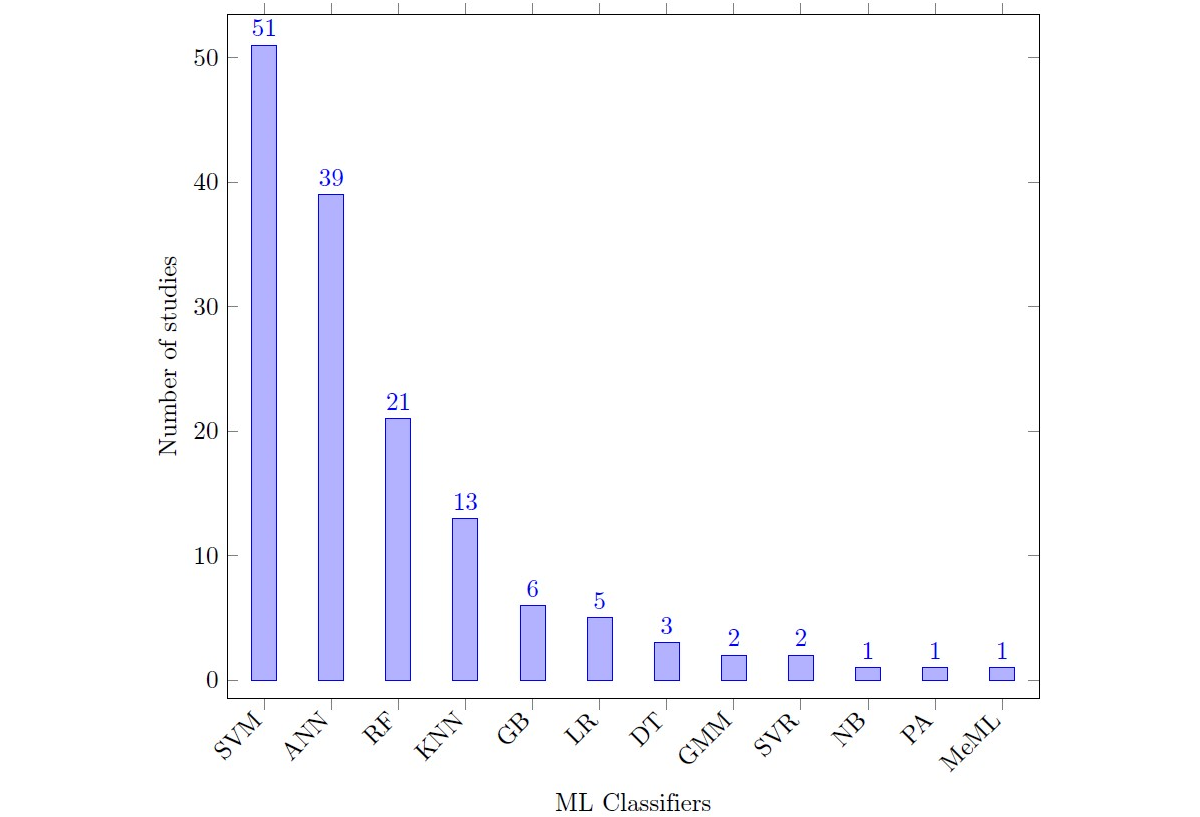
The usage of machine learning algorithms. ANN: artificial neural network; DT: decision tree; GB: gradient boosting; GMM: Gaussian mixture model; KNN: K-nearest neighbor; LR: logic regression; MeML: mixed effect machine learning; ML: machine learning; NB: naïve Bayes; PA: passive active; RF: random forest; SVM: support vector machine; SVR: support vector regression.

The usage of the 12 ML techniques is shown in Figure 2, where the support vector machine algorithm was the most used (51/145, 35.2%) and artificial neural networks were the second most utilized technique (39/145, 26.9%) among all ML techniques. Several studies have tested and compared different algorithms. Figure 2 shows the ML technique with the best results from each study. The support vector machine notation contains all different kernel combinations, and all utilized neural network architectures are grouped under artificial neural network. As many as 11 of the 12 ML techniques shown in Figure 2 have tested on PD, 5/12 on AD, 4/12 on CI/MCI, 5/12 on COVID-19, 3/12 on ALS, 1/12 on cardiovascular disorders, 1/12 on essential tremor, 2/12 on multiple sclerosis, 1/12 on stroke, 1/12 on asthma, 1/12 on autism, 1/12 on fatigue, 1/12 on chronic obstructive pulmonary disease, 1/12 on NCC, 1/12 on functional dysphagia/oropharyngeal dysphagia, 2/12 on depression, 1/12 on influenza disease, 1/12 on ND, and 1/12 on traumatic brain injury (Table 2).

### Time and Geographical Trend of the Publications

**Figure 3:**
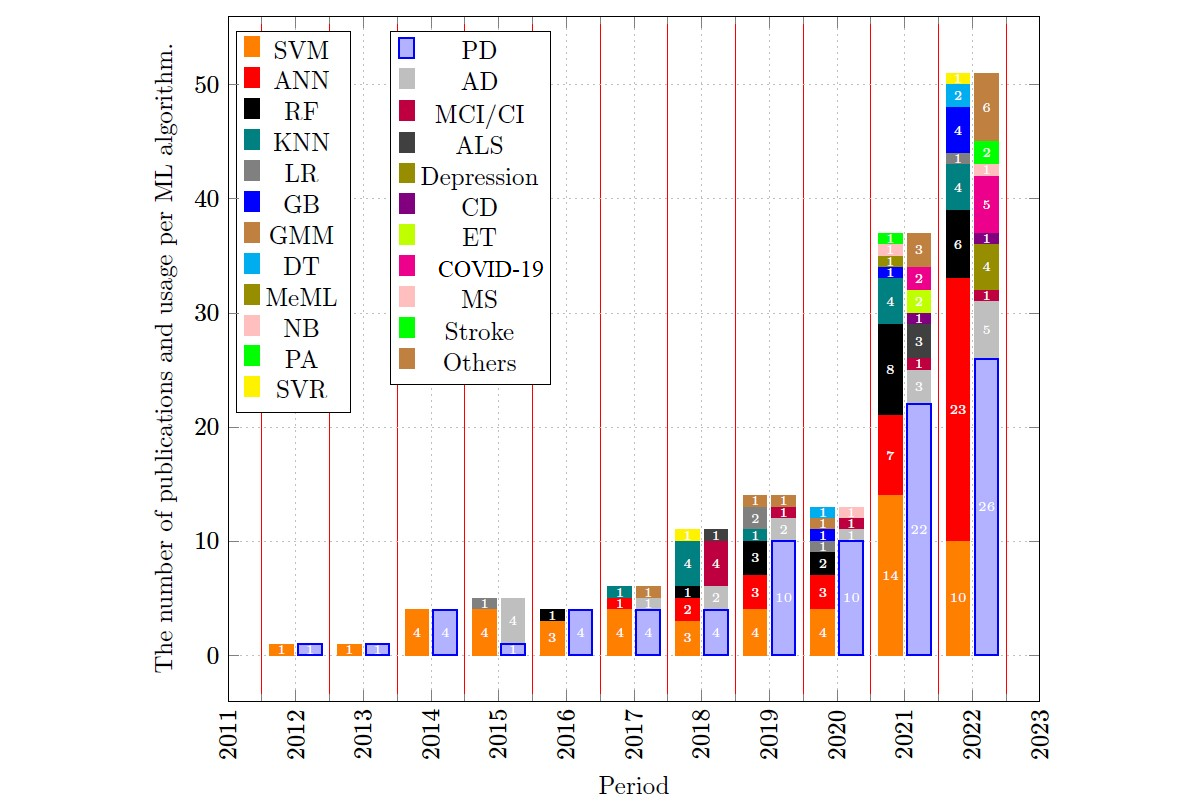
Usage of ML techniques and investigated disorders by year. AD: Alzheimer disease; ALS: amyotrophic lateral sclerosis; ANN: artificial neural network; CD: cardiovascular disease; CI: cognitive impairment; DT: decision tree; ET: essential tremor; GB: gradient boosting; GMM: Gaussian mixture model; KNN: K-nearest neighbor; LR: logic regression; MCI: mild cognitive impairment; MeML: mixed effect machine learning; ML: machine learning; MS: multiple sclerosis; NB: naïve Bayes; PA: passive active; PD: Parkinson disease; RF: random forest; SVM: support vector machine; SVR: support vector regression.

Figure 3 shows the published studies by year and the investigated disorders. The results indicate that there is an upward trend in the studies involving the application of ML for voice-affecting disorder. Up to 2016, the focus of the research was solely on PD and AD. In the last 5 years, the research on voice-based diagnosis and monitoring with ML has not only increased but also diversified in terms of the investigated voice-affecting disorders with the addition of CI/MCI, ALS, cardiovascular disorder, essential tremor, COVID-19, multiple sclerosis, NCC, functional dysphagia/oropharyngeal dysphagia, depression, influenza disease, ND, stroke, asthma, autism, fatigue, chronic obstructive pulmonary disease, and traumatic brain injury. In addition, the highest publication rate occurred in 2022 (more than doubled compared with previous years); 51/145 studies included in this SLR have been published in 2022, which corresponds to 35.1% of all listed articles in Multimedia Appendix 1.

**Figure 4:**
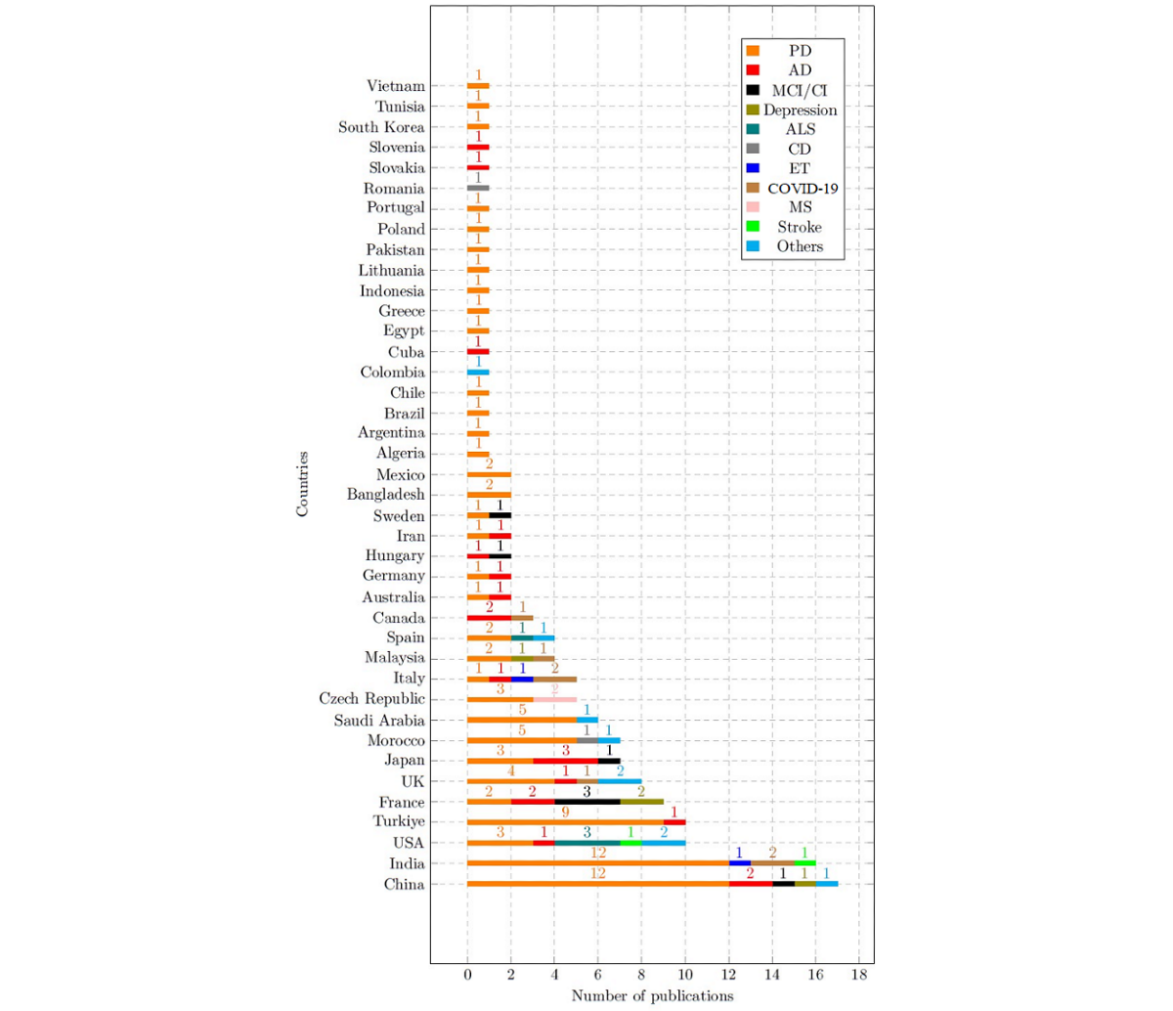
Investigated disorders by country. AD: Alzheimer disease; ALS: amyotrophic lateral sclerosis; CD: cardiovascular disorder; CI: cognitive impairment; ET: essential tremor; MCI: mild cognitive impairment; MS: multiple sclerosis; PD: Parkinson disease.

Figure 4 displays the contribution from countries for a specific disorder. Some countries tend to focus more on 1 disorder, while others investigated several voice-affecting disorders using ML techniques. In addition, PD seems to be the most investigated disorder for the majority of countries. The geographical trend described in this section reflects the country in which the study was performed and not the geographical source of the sample.

### Data Characteristics

This section describes the characteristics of voice and nonvoice data used as input into the ML models. A total of 11/145 (7.6%) studies integrated nonvocal data in conjunction with vocal features to form the input feature sets for the ML models; 6 of these studies [54,66,96,105,149,163] incorporated demographic data, including gender, age, BMI, comorbidities, weight, height, and disease duration. Meanwhile, 2 of the studies [141,153] used video inputs, while 3 studies [83,158,159] incorporated external sensor signals such as electromyography and motion trackers.

Table 3 compiles the disorders and frequency of recorded data characteristics with the density of extracted vocal features and data source. Results indicate that vowel phonations are one of the most adopted recording types among almost all listed disorders. A total of 68 studies chose to base their analysis on vowel recordings, 33 combining different recordings, 20 free speeches, 12 scripted speeches, 9 picture descriptions, and 3 studies used syllable recordings. Cognitive disorders (eg, AD and MCI) that tend to use voice features extracted from speech and other disorders (eg, PD) lean toward features extracted from vowel phonation.

In all studies, raw data that consist of recordings underwent signal processing to extract features that were used as input data into ML techniques. Identified signal processing implementations were baseline acoustic (BLA), Mel-frequency cepstral coefficients, tunable Q-factor wavelet transform, wavelet transform, and spectrogram, which are frequency-transformed versions of the input signal that generates features in the form of digits and images. Other utilized features were linguistic and vocal features that generate statistical outputs (eg, silence rate, pause rate, duration, and ineligibility). In addition, combining several vocal features is more popular than only using BLA features, where almost 69/145 (47.6%) of the studies combined several features as input. But still, BLA features (36/145, 24.8%) are one of the most separately used feature sets. In this SLR BLA corresponds to all or a portion of acoustic, time, and frequency domain features calculated from raw recordings (eg, pitch, zero cross rate, jitter, shimmer, and formant frequencies).

**Table 3:** Characteristics of the input data and data source.

Disorder	Recording	Feature	Data source
Parkinson disease	Vowel: 59 [17,40,42,44,48,50,54–59,61–64, 68,69,74–85,87–91,93–97,99,100,102,119–122, 124,126–128,130–132,135,170,183,184,192] Combined: 20 [49,51,53,60,65,66,71, 73,98,101,118,123,125,133,134,171,180,181,185,188] Scripted speech: 3 [52,72,117] Free speech: 3 [70,92,129] Syllable: 2 [67,86]	BLA^a^: 26 [17,40,50,53,56, 58-60,64-66,71,73,77,79,82,84, 87,88,92,98,118,132,133,184,188] Combined: 45 [42,44,48,51,52,54, 57,62,63,67–70,72,74,76,79,81,85, 86,89–91,93,94,96,97,99,100,119,120,122, 124,126–131,134,135,170,171,184,192] MFCC^b^: 5 [49,61,80,94,101] Spectrogram: 7 [75,117,123,125,180,181,185] RP^c^: 1 [95] TQWT^d^: 2 [55,121] WT^e^: 1 [83]	CFS^f^: 31 [48,51,52,65,67, 70,72-74,78-81,83,85,86,88, 92,94,96,97,101,102,117, 130,131,170,171,180,185] UCI^g^: 33 [40,42,44,50, 53,55–57,60,61,63,64,68,69,71,82, 84,91,93,99,100,119,120,122, 124,126,127,129,133,135,183,188,192] Multiple: 14 [49,54,58,59, 66,75,87,90,98,118,123,125, 132,181] mPower: 4 [17,62,77,128] NCVS^h^: 1 [88] PARCZ^i^: 1 [89] NG^j^: 3 [95,121,134]
Dementia, Alzheimer disease	Free speech: 8 [103,105,108,172,173,175] Picture description: 7 [109,110,136-139,174] Scripted speech: 2 [104,176] Combined: 1 [179]	Combined: 11 [103,104,107,109,110,136,138,139,173-175] Vocal: 2 [105,172] BLA: 2 [176,179] Spectogram: 2 [108,137] Speech statistic: 1 [106]	CFS: 10 [103-108,172,173,175,176] ADBC^k^: 7 [109,110,136-139,174] Multiple: 1 [179]
Cognitive impairment/mild cognitive impairment	Free speech: 3 [112,114,115] Picture description: 2 [116,178] Scripted speech: 2 [113,177] Combined [150]	BLA: 3 [113,114,177] Combined: 2 [112,116] Linguistic: 1 [115] Vocal: 1 [178] Spectogram [150]	CFS: 8 [112-116,150,177,178]
COVID-19	Vowel: 2 [156,157] Combined: 5 [143-147]	Combined: 3 [144,156,157] Spectogram: 3 [143,145,146] MFCC [147]	Coswara: 4 [143,144,156,157] Multiple: 1 [146] CFS: 1 [145] CHRSD^l^: 1 [147]
Amyotrophic lateral sclerosis	Vowel: 2 [46,160] Scripted speech: 1 [159] Combined: 1 [158]	BLA: 2 [46,160] Combined: 2 [158,159]	CFS: 4 [46,158-160]
Depression	Free speech: 2 [141,161] Scripted speech [140] Combined [142]	Combined: 2 [142,161] Spectrogram: 2 [140,141]	Multiple: 2 [141,161] CFS: 2 [140,142]
Cardiovascular disorders	Vowel: 1 [111] Combined: 1 [186]	BLA: 1 [111] MFCC: 1 [173	CFS: 2 [111,186]
Essential tremor	Vowel: 2 [162,163]	BLA: 1 [162] Spectrogram: 1 [163]	CFS: 2 [162,163]
Multiple sclerosis	Syllable [164] Scripted speech [149]	Spectrogram [164] BLA [149]	CFS: 2 [149,164]
Stroke	Combined: 2 [148,153]	MFCC [148] Spectrogram [153]	CFS: 2 [148,153]
Autism	Free speech [151]	Spectrogram [151]	CFS: 1 [151]
Asthma	Free speech [182]	BLA [182]	CFS: 1 [182]
Fatigue	Scripted speech [187]	Combined [187]	CFS: 1 [187]
Chronic obstructive pulmonary disease	Scripted speech [154]	BLA [154]	CFS: 1 [154]
Neurodegenerative cognitive complaint	Free speech [165]	Combined [165]	CFS: 1 [165]
Functional dysphagia/oropharyngeal dysphagia	Combined [166]	Combined [166]	CFS: 1 [166]
Influenza disease	Vowel [167]	WT [167]	CFS: 1 [167]
Neurological disease	Vowel [169]	Combined [169]	CFS: 1 [169]
Traumatic brain injury	Free speech [152]	Spectrogram [152]	TBIBank^m^ Coelho corpus: 1 [152]

^a^BLA: baseline acoustic.

^b^MFCC: Mel-frequency cepstral coefficients.

^c^RP: recurrence plot.

^d^TQWT: tunable Q-factor wavelet transform.

^e^WT: wavelet.

^f^CFS: collected for study.

^g^UCI: University of California, Irvine.

^h^NCVS: National Center for Voice and Speech.

^i^PARCZ: Czech Parkinsonian Speech Database.

^j^NG: not given.

^k^ADBC: Alzheimer Dementia Bank blog corpus.

^l^CHRSD: Corona Hack Respiratory Sound data set.

^m^TBIBank: Traumatic Brain injury bank.

A total of 70 studies collected data for a specific study and 75 studies gathered data from an available data set. Sakar et al (2013) [73] and Sakar et al (2019) [48] are 2 different data sets donated to the UCI (University of California, Irvine), which have been used in 15 different included studies in this SLR; 5 studies [53,57,60,71,100] used the UCI data set containing 20 participants with PD and 20 HC participants, and 15 studies [40,42,44,48,55,61,64,68,99,119,121,122,124,126,127] used the UCI data set having 188 participants with PD and 64 HC participants from the same source. UCI and Coswara provide data sets that can be accessed and downloaded without any additional application [73,189]. All other data sources identified in this SLR require an application or are not publicly available. Data sets used in studies are unbalanced. Even if there is equality between the number of participants in terms of disordered and HC groups, a closer inspection of data sets reveals gender inequality. For example, Sakar et al (2013) [73] included 20 participants with PD and 20 HC participants; however, a closer inspection showed that the PD group comprised 6 females and 14 males, and the HC group consisted of 10 females and males, respectively. Another issue is the low number of participants in studies, where only 8/145 studies [17,62,77,78,81,92,113,170] based their outcome on more than 100 participants for both pathological and HC groups at the same time.

### Observation Time

SQ5 aims to find out whether studies rely on longitudinal data and observation over time or observations at the same time that study was done. As the authors predefine the participants and measure the exposures and outcomes at the same time in all included studies, all studies in this SLR follow the cross-sectional study design [190].

### Performance Evaluation

Measures presented to assess the efficiency of the ML techniques used show diversity in the included articles. Accuracy is one of the most used measures to present the outcome of almost all studies. Sensitivity, specificity, precision, Matthew’s correlation coefficient, area under the curve, *F*_1_-score, recall, mean absolute error, *R*^2^, positive predictive value, and negative predictive value were other used measures in combination with accuracy without any standard order. Under the *performance* column in Multimedia Appendix 1, all combinations can be seen; 7 articles, 5 from the PD group [63,89,97,170,171], 1 from the AD group [172], and 1 from the ALS group [114], have presented results discriminated by gender and only 1 study [92] paid attention to language differences.

Two groups of studies [48,73] from UCI data sets were found to be suitable for meta-analysis due to the homogeneity between studies. The first group consisted of 15 studies using a data set containing voice recordings from 188 participants with PD and 64 HC participants [40,42,44,48,55,61,64,68,99,119,121, 122,124,126,127]. The second group consisted of 5 studies [53,57,60,71,100] using voice recordings from 20 participants with PD and 20 HC participants (Table 4). Studies employing the first data set achieved 0.925 average accuracy within an accuracy range of 0.790-0.997. Studies employing the second data set achieved 0.869 average accuracy within an accuracy range of 0.670-0.990.

**Table 4:** List of comparable studies.

Data set^a^	Classifier	Feature	Performance	Reference
CFS^b^ (donator)	SVM^c^	MFCC^d^ and TQWT^e^	Accuracy: 0.8600	[48]
UCI^f^	GB^g^	BLA^h^ and spectrum	Accuracy: 0.9388	[44]
UCI	KNN^i^	TQWT	Accuracy: 0.9800	[55]
UCI	ANN^j^	BLA	Accuracy: 0.9921	[40]
UCI	SVM	BLA, MFCC, WT^k^, and TQWT	Accuracy: 0.9160	[42]
UCI	ANN	MFCC	Accuracy: 0.9674	[61]
UCI	NB^l^	BLA	Accuracy: 0.7897	[64]
UCI	SVM	BLA, MFCC, TQWT, and WT	Accuracy: 0.9470	[68]
UCI	SVM	BLA, MFCC, WT, and TQWT	Accuracy: 0.9350	[99]
UCI	SVM	BLA, MFCC, and TQWT	Accuracy: 0.8660	[119]
UCI	KNN	TQWT	Accuracy: 0.9890	[121]
UCI	RF^m^	BLA and MFCC	Accuracy: 0.8884	[122]
UCI	ANN	BLA, MFCC, and TQWT	Accuracy: 0.9200	[124]
UCI	SVM	BLA, MFCC, TQWT, and WT	Accuracy: 0.9621	[126]
UCI	ANN	BLA, MFCC, and TQWT	Accuracy: 0.9974	[127]
*UCI*	SVM	BLA	Accuracy: 0.6701	[53]
*UCI*	RF	BLA and MFCC	Accuracy: 0.9433	[57]
*UCI*	ANN	BLA	Accuracy: 0.9903	[60]
*UCI*	ANN	BLA	Accuracy: 0.8647	[72]
*UCI*	SVM	BLA and MFCC	Accuracy: 0.8750	[100]

^a^Italicized data sets represent Parkinson disease data set 2 containing data on patients with Parkinson disease (n=20) and HC (n=20); all other data sets correspond to Parkinson disease data set 1 containing data on patients with Parkinson disease (n=188) and HC (n=64).

^b^CFS: collected for study.

^c^SVM: support vector machine.

^d^MFCC: Mel-frequency cepstral coefficients.

^e^TQWT: tunable Q-factor wavelet transform.

^f^UCI: University of California, Irvine.

^g^GB: gradient boosting.

^h^BLA: baseline acoustic.

^i^KNN: K-nearest neighbor.

^j^ANN: artificial neural network.

^k^WT: wavelet.

^l^NB: naïve Bayes.

^m^RF: random forest.

## Discussion

### Principal Findings

In this SLR, 10 years of research on ML techniques applied for diagnosing and monitoring voice-affecting disorders indicates an extended interest from many countries. It seems that researchers have focused mostly on the detection of 19 identified disorders with low number of individuals in data sets that lead to gaps identified as the main findings of this SLR. These are summarized below:

- Most studies aimed to perform a diagnostic test through the detection or classification of disorders, and only a few studies aimed to monitor a specific disorder.
- PD was the most investigated disorder among all 19 voice-affecting disorders.
- There was a broad interest from many counties.
- Data sets used in studies were unbalanced, and most studies collected their data without providing open access. Additionally, only 11/145 (7.6%) included studies considered using additional data in conjunction with voice features.
- All studies were cross-sectional.
- Accuracy was the most common metric for the overall performance evaluation.

The majority of the studies focused on the detection or classification of the 19 identified voice-affecting disorders through emerging ML techniques. However, it is important to also consider the need for continuous monitoring of these disorders to improve the quality of life for those affected. Another consequence of focusing solely on detection is that it may not provide enough information about the severity of the disorder, which is a vital measure for decision-making on treatment or determining correct dosage for medication. Therefore, to improve the applicability of findings in clinical practices, it may be beneficial to navigate the focus of research toward methods for monitoring the progression, which involve severity measures of voice-affecting disorders.

Verdolini et al [6] give an intuition that the 19 disorders identified in this SLR correspond only to a small number of voice-affecting disorders that have been studied in research. This small correspondence makes it troublesome to highlight the digital biomarkers that are specifically related to a single disorder, which is essential for distinguishing underlying conditions that lead to altered voice quality. To address this issue, it is worth extending the research to other voice-affecting disorders that have been underrepresented in previous studies. This would not only extend the number of disorders being studied but also allow for the identification of differences and similarities in terms of digital biomarkers or other features across a wider range of disorders. Exploring the differences and similarities between disorders, syndromes, and symptoms is also beneficial because some disorders can function as symptoms of other underlying conditions affecting voice production, that is, while depression can be considered a disorder in and of itself, it can also manifest as a symptom of PD [6].

Based on the origin of the publication and the origin of the data sets, a wide interest from many countries was observed. However, many countries conduct research on the same data sets, which can lead to both positive and negative results regarding the clinical applicability of outcomes. Concentration on a group of data sets may increase the performance of the ML technique for the represented input data attributes. By contrast, it may also introduce limitations for the nonrepresented or underrepresented data. For example, the UCI data set in Sakar et al [73] contains several voice recordings in Turkish; using this data set may give satisfying results for recordings in the same language, but using it on English recordings could be problematic. However, interest from many countries shows enormous potential for collecting more available data sets and generalized ML techniques.

A balanced data set means the numbers of samples are relatively equal between classes, giving equivalent contributions from all classes during training, which eventually improves the performance of the ML technique on new data. By contrast, imbalanced data can lead to bias. The results of our SLR show that using balanced data has not been considered in studies. As the voice is used as a medium to detect a disorder, it is important to consider the effect of linguistic diversity, gender, age, and other sociodemographic differences on the generalizability of a system. Training and testing an ML technique on balanced data offer higher reliability for use in clinical practices. Balancing data based on different characteristics may be another option for higher reliability (eg, only male or only female). The studies included in the analysis provided demographic information about their respective data sets. However, only a limited number of studies incorporated this information into the vocal feature set that use additional nonvoice data for training the ML models. Integrating the demographic data into the automated process of data set preparation could prove beneficial, as opposed to the manual preparation of data sets based on disparate attributes. Additionally, combining multiple sensory inputs along with vocal features may further enhance the performance of the ML algorithms. However, this practice appears to be infrequently observed in recent studies.

Results of this SLR showed that 70/145 (48.3%) studies collected data specific to the research without making them publicly available. It is observable that PD is one of the most investigated disorders. That might be a result of publicly available data obtained from the UCI Parkinson data set repository. It is worthwhile to extend publicly available data sources with varied voice-affecting disorders and features to preserve research reliability and homogeneity in the scope. Another aspect that would influence clinical applicability is the small number of participants being considered in the research. Increasing the number of participants might increase the reliability of ML techniques.

In SLRs, “longitudinal study” refers to a recurrent sample taken from the same participant over time, which is a way of following the progression and trend of a disorder that helps to identify the patterns and causal relationships. Conducting a longitudinal study may even help to reduce the confounding variables [191].

The absence of longitudinal studies makes it difficult to conduct an epidemiological trend analysis of time effects on digital features extracted for ML techniques to diagnose and monitor voice-affecting disorders. All included studies in this SLR considered cross-sectional analysis, which does not represent the possible divergence tied to the progression of a specific disorder and individual. Therefore, longitudinal studies are essential to discover the voice changes over time [191,192].

The majority of the studies chose to represent the performance in accuracy, specificity, and sensitivity metrics, which were tied to the overall classification performance in research on voice-affecting disorder diagnosis and monitoring. In this SLR, only 8 results indicated that gender and language diversity may affect the performance in terms of accuracy [63,89,92,97,114,170-172]. As none of the studies address the effect of unbalanced data on performance evaluation, it is noteworthy that the divergence in accuracy in those outcomes could be the effect of unbalanced data. However, different accuracy results may be due to many other aspects. Regardless of the employed ML technique, used features; number of features; the proportion between training, validation, test sets; and feature extraction techniques may also be a factor in deviating accuracy results.

### Limitations

The decision to include studies published only in English is a risk of missing important evidence in other languages, which at the same time is an unavoidable limitation for the generalization of this SLR. Another factor that can be considered as a limitation is only including peer-reviewed studies, which do not consider conference papers. Relying on the conference papers can be problematic due to the limitations including the potential for incomplete or preliminary results [193]. Additionally, including low-quality studies may introduce a risk of bias, as these studies may have suffered from selective reporting bias. In the SLR presented herein, this risk was mitigated by checking beyond what is presented in the paper, that is, when the methodological information was referenced elsewhere, the authors checked and considered the referenced material when conducting the quality assessment. Additionally, during the screening phase, when the abstract did not contain the full information to fulfill the inclusion criteria, these pieces were marked as “maybe” cases that were checked further before being fully read.

### Future Work

Underrepresented monitoring purposes, research on a low number of voice-affecting disorders, unbalanced data, limited public voice data, lack of longitudinal research, and performance evaluation without paying attention to diversities were 6 gaps addressed in this SLR, which may be considered in future research. We suggest the following:

- One research direction may be to include disorders that were underrepresented in the state of the art. It is essential to take the gaps into consideration, such as working with balanced and extended data sets, to generate more reliable results.
- Conducting cross-sectional and longitudinal studies to identify specific digital features that are associated with voice-affecting disorders can be beneficial for determining the severity of the disorder and monitoring it over time.
- Studying the effects of demographic characteristics, such as gender, age, linguistic factors, and other relevant additional data on the classification models may also provide insights for building more accurate ML techniques for specific disorders.

### Conclusions

Through the methodology of an SLR, we identified 145 studies on the use of voice for diagnosing or prognosing disorders, by the means of ML algorithms. These studies were summarized in terms of many aspects, including disorders and conditions that affect the voice, characteristics of the input data, ML techniques used for voice-based diagnosis, and research interests from countries. The findings of this SLR indicated that most of the studies are concerned with the detection and classification of investigated disorders and conditions based on cross-sectional studies. This study also found gaps in the literature, such as the usage of unbalanced data sets, lack of longitudinal studies, research not addressing nonvoice data in the voice studies, and most voice-affecting disorders in the interest of this study being underrepresented in research. Research in the field of voice-based diagnostics with the utilization of ML is making the practical application of this technology in health care more achievable. The use of voice as a digital biomarker could open the possibilities to large population screening of many disorders in a low-cost, noninvasive, and scalable way. To implement such a system in a clinical setting, the exploration of unknown aspects is an essential process to proceed with. To do that, it is necessary to extend the research on all possible voice-affecting disorders and identify the nuances between all different voice-affecting disorders and their effect on vocal features. Currently, research in this field primarily focuses on detection using a limited number of participants. However, for more generalizable results in the future, research may not only consider increasing the participant numbers but also maintaining a balance among them and identifying the measures that can be used for monitoring purposes.

There is a broad research interest from many countries, which creates a potential for observing the effects of cultural and language differences on ML algorithms. However, contribution to data collection and increasing the size of available data with diverse characteristics are crucial steps that each country might consider.

## Appendix

Multimedia Appendix 1 Summary of included studies.

Multimedia Appendix 2 PRISMA (Preferred Reporting Items for Systematic Reviews and Meta-Analyses) 2020 checklist.

Multimedia Appendix 3 PRISMA (Preferred Reporting Items for Systematic Reviews and Meta-Analyses) 2020 for Abstract checklist.

## Abbreviations

AD: Alzheimer disease
ADBC: Alzheimer Dementia Bank blog corpus
ALS: amyotrophic lateral sclerosis
ANN: artificial neural network
BLA: base line acoustic
CI: cognitive impairment
CFS: collected for study
CHRSD: Corona Hack Respiratory Sound data set
DT: decision tree
GB: gradient boosting
GMM: Gaussian mixture model
HC: healthy control
KNN: K-nearest neighbor
LR: logic regression
MCI: mild cognitive impairment
MeML: mixed effect machine learning
MeSH: Medical Subject Headings
MFCC: Mel-frequency cepstral coefficients
ML: machine learning
NB: naïve Bayes
NCC: neurodegenerative cognitive complaint
NCVS: National Center for Voice and Speech
ND: neurological disease
NG: not given
PA: passive active
PARCZ: Czech Parkinsonian Speech Database
PD: Parkinson disease
PICO: population, intervention, comparison, and outcome
PRISMA: Preferred Reporting Items for Systematic Reviews and Meta-Analyses
RF: random forest
RP: recurrence plot
SLR: systematic literature review
SQ: subquestion
SVM: support vector machine
SVR: support vector regression
TBIBank: Traumatic Brain injury bank
TQWT: tunable Q-factor wavelet transform
UCI: University of California, Irvine

## Data Availability

A summary table of all included studies and extracted data is available as Multimedia Appendix 1.
