# Supplementary material for: Applied Machine Learning Techniques to Diagnose Voice-Affecting Conditions and Disorders: Systematic Literature Review
Source: J Med Internet Res. 2023 Jul 19;25:e46105. doi: 10.2196/46105 (PMC10398366; doi:10.2196/46105)
Supplement: Multimedia Appendix 1 [file jmir_v25i1e46105_app1.pdf]

All included studies

Table 8. Summary of included studies.

Nr	Ref.	Dataset	Recording	Subjects	Classifier	Feature	Aim	Performance
				Parkinson's Disease (PD)				
1	[1]	CFS	Vowel	PD(180), HC(64)	SVM-RBF	MFCC, TQWT	Calssification	Accuracy: 0.86, F1-score: 0.84, MCC: 0.59
2	[2]	Neurovoz, GITA, CzechPD, Albayzin FisherSP	Syllable, Free Speech, Sentence	PD(117), HC(96)	fGMM-fUBM	MFCC	Detection	Accuracy: 0.94, AUC: 0.97, Sensitivity: 0.9
3	[3]	CFS	Vowel	PD(26), HC(16)	RF	BLA	Detection	Accuracy: 0.9566, Sensitivity: 0.9077, Specificity: 0.9810
4	[4]	CFS	Sentence	PD(30), HC(15)	LSTM	BBEs, MFCC, DMFCC, DDMFCC	Detection	Accuracy: 0.8429, F1-score: 0.8852, Sensitivity: 0.8734, Specificity: 0.9111, MCC: 0.6603
5	[5]	CFS	Scripted Speech	PD(11), HC(29)	CNN	TQWT-RSSD-PSD based image	Diagnosis	Accuracy: 0.9937
6	[6]	UCI	Vowel	PD(188), HC(64)	XGBoost	BLA, Spectrum	Detection	Accuracy: 0.9388, F1-score: 0.9374, Sensitivity: 0.9166, Specificity: 0.9609, AUC 0.978, Precision 0.9591
7	[7]	UCI	Vowel	PD (42)	ISVR	BLA	Prediction of progression	Total-MAE: 0.4656 motor-MAE:0.4967
8	[8]	UCI	Vowel, Sentence, Words, Number count	PD(20), HC(20)	SVM	BLA	Diagnosis	Accuracy: 0.6701, Sensitivity: 0.7615, Specificity: 0.5788 MCC: 0.34
9	[9]	UEX, mPower	Vowel	PD(60), HC(60)	PA	BLA, MFCC	Voice condition analysis	Accuracy: 0.9205, Sensitivity: 0.9396, Specificity: 0.9018 AUC: 0.9756
10	[10]	UCI	Vowel	PD(188), HC(64)	IG-KNN	TQWT	Diagnosis	Accuracy: 0.98
11	[11]	UCI	Vowel	PD(23), HC(8)	BFO-SVM	BLA	Prediction	Accuracy: 0.9742, Sensitivity: 0.9929, Specificity: 0.9150
12	[12]	UCI	Vowel	PD(188), HC(64)	NN	BLA	PD and gender recognition	Accuracy: 0.9921. F1-score: 0.9921
13	[13]	UCI	Vowel	PD(20), HC(18	RF	BLA, MFCC	Detection	Accuracy: 0.9433, Precision: 0.9440, F1-score: 0.9430
14	[14]	UCI	Vowel	PD(188), HC(64)	SVM	BLA, MFCC, WT, TQWT	Classification	Accuracy: 0.916. F1-score: 0.946, MCC: 0.772
15	[15]	UCI + CFS	Vowel	PD(113), HC(28)	SVM	BLA	Classification	Accuracy: 0.8874, Precision: 0.8889, Recall: 0.9703 F1-score: 0.9255 MEA: 3.7699
16	[16]	Oxford + Istanbul	Vowel	PD(71), HC(28)	CBFO-FKNN	BLA	Diagnosis	Accuracy: 0.9697, Sensitivity: 0.9687, Specificity: 0.9875 AUC: 0.9781
17	[17]	UCI	Vowel, Sentence, Words, Number count	PD(20), HC(20)	LSTM	BLA	Detection	Accuracy: 0.9903
18	[18]	UCI	Vowel	PD(188), HC(64)	ELM	MFCC	Classification	Accuracy: 0.9674
19	[19]	mPower	Vowel	PD(1087), HC(5581)	XGBoost	BLA, MFCC	Detection	Accuracy: 0.0.9578
20	[20]	UCI	Vowel	PD(64), HC(64)	KNN	BLA, MFCC, TQWT, WT	Detection	MALE -> Accuracy: 0.9590, Sensitivity: 0.9835, Specificity: 0.9106 Precision: 0.9560 / FEMALE -> Accuracy: 0.9436, Sensitivity: 1.0000, Specificity: 0.9710 Precision: 0.9683
21	[21]	UCI	Vowel	PD(188), HC(64)	NB	BLA	Detection	Accuracy: 0.7897, Precision: 0.9260
22	[22]	CFS	Vowel, Sentence, Words, Number count	PD(48), HC(20)	NN	BLA	Detection	Accuracy: 1.0000, Sensitivity: 1.0000, Specificity: 0.9500
23	[23]	CFS(HC), UCI(PD)	Vowel, Free speech	PD(30), HC(30)	RF	BLA	Detection	Accuracy: 0.9994
24	[24]	CFS	Syllable	PD(24), HC(22)	SVM	AF	Evaluation of Articulatory Disorders in PD	Accuracy: 0.8800
25	[25]	UCI	Vowel	PD(188), HC(64)	SVM	BLA, MFCC, TQWT, WT	Detection	Accuracy: 0.9470, Sensitivity: 0.9840, Specificity: 0.9268, Precision: 0.9722, False Alarm Rate: 0.0277, MCC: 0.8686, F1-score: 0.9633
26	[26]	UCI	Vowel	PD(48), HC(20)	RF	BLA	Detection	Accuracy: 100
27	[27]	CFS	Free speech	PD(60), HC(20)	SVM	BLA, MFCC	Classification speech ineligibility	Accuracy: 0.9200, AUC: 0.9100
28	[28]	UCI	Vowel, Scripted speech	PD(20), HC(20)	ANN	BLA	Detection	Accuracy: 0.8647, Sensitivity: 0.8891, Specificity: 0.8402, MCC: 0.07321
29	[29]	CFS	Scripted speech	PD(40), HC(40)	SVM	AF, PF, BLA	Dimension identification	Accuracy (PD vs HC): 0.8400, Accuracy (PD-MCI vs HC): 0.8690, Accuracy (PD vs PD-MCI): 0.7210
30	[30]	CFS	Vowel, Sentence, Words, Number count	PD(48), HC(20)	SVM	BLA	Detection	Accuracy: 0.8500, Sensitivity: 0.8000, Specificity: 0.9017, MCC: 0.07035

Table 8. Summary of included studies.

Nr	Ref.	Dataset	Recording	Subjects	Classifier	Feature	Aim	Performance
31	[31]	CFS	Vowel	PD(24), HC(22)	SVM	AF, PF, BLA	Discrimination	Accuracy: 0.8100, AUC: 0.8400
32	[32]	SVD, PC-GITA	Vowel	PD(50), HC(50)	CNN	Spectrogram	Detection	Accuracy: 0.9000
33	[33]	CFS	Vowel	PD(22), HC(22)	SVM	Spectrogram, LFCC, GTCC, MFCC	Detection	Accuracy: 0.8640, Sensitivity: 0.9700, Specificity: 0.9320
34	[34]	mPower	Vowel	Depressed PD(318), Nondepressed PD(603)	RF	BLA	Depression screening	Accuracy: 0.7700, Precision: 0.7200, Recall: 0.5600 F1-score: 0.6300
35	[35]	CFS	Vowel	PD(262), HC(464)	XGBoost	BLA, MFCC	Detection	Accuracy: 0.7400, AUC: 0.7530
36	[36]	CFS	Vowel	PD(64), HC(35)	KNN	BLS, MFCC, TQWT, WT	Detection	Accuracy: 0.9455, Sensitivity: 0.9455, Specificity: 0.9426, AUC: 0.8700
37	[37]	CFS	Vowel	PD(17), HC(17)	SVM	MFCC	Detection	Accuracy: 0.9118, Sensitivity: 1.0000, Specificity: 0.8235
38	[38]	CFS	Vowel	PD(1483), HC(8300)	RF	BLA, MFCC	Assessment	Accuracy: 0.6879, Sensitivity: 0.6490, Specificity: 0.6796
39	[39]	UCI	Vowel	PD(23), HC(8)	SVM	BLA	Dysphonia Detection	Accuracy: 0.9180
40	[40]	CFS	Vowel	PD(50), HC(15)	ELM	WT	Severity	Accuracy: 0.9553
41	[41]	UCI	Vowel	PD(23), HC(8)	SVM	BLA	Identification	Accuracy: 0.9900, Sensitivity: 0.9900, Specificity: 1.0000
42	[42]	CFS	Vowel	PD(30), ND(20), HC(50)	SVM	MFCC	Classification	Accuracy PD vs ND: 0.9000, Accuracy PD vs HC: 0.9600
43	[43]	mPower	Vowel	PD(246), HC(2023)	DT	BLA	Detection	AUC: 0.9500. F1-score: 0.8360, Precision: 0.9010, Recall: 0.797
44	[44]	CFS	Syllable	PD(30), HC(30)	KNN	Energy Entropy, Energy, Zero Crossing Rate, Spectral Rolloff, Spectral Centroid, Spectral Flux	Detection	Accuracy: 0.8230, Sensitivity: 0.8800
45	[45]	UCI, SVD	Vowel	PD(23), HC(39)	SVM, ELM	BLA	Monitoring	Accuracy: 0.9720
46	[46]	NCVS	Vowel	PD(33), HC(10)	SVM	BLA	Classification	Accuracy: 0.9900
47	[47]	CFS	Vowel	PD(14)	SVM	BLA, MFCC	Treatment assessment	Accuracy: 0.90
48	[48]	PARCZ	Vowel	PD(91), HC(51)	ANN	AKV	Detection	MALE -> Accuracy: 0.9945, Sensitivity: 0.9946, Specificity: 0.9944 / FEMALE -> Accuracy: 0.9942, Sensitivity: 0.9942, Specificity: 0.9941 Precision: 0.9683
49	[49]	UCI, CP-PDD	Vowel	PD(65), HC(68)	SVM	IMFCC, TQWT	Detetction	Accuracy: 0.9654
50	[50]	UCI	Vowel	PD(25), HC(20)	RF	BLA. IMFCC	Prediction	Accuracy: 1.0000, AUC: 0.9900
51	[51]	CFS	Free speech	PD(106), HC(392)	SVM	BLA	Detection	English -> AUC:0.8400, German -> AUC:0.8300, Greek -> AUC:0.9300, All -> AUC:0.8200
52	[52]	UCI	Vowel	PD(188), HC(64)	GMM	BLA, MFCC, WT, TQWT	Detection	Accuracy: 0.8912 MCC: 0.7060
53	[53]	CFS	Vowel	PD(60), HC(100)	SVM	MFCC	Diagnosis	Accuracy: 0.9750 Sensitivity: 1.0000, Specificity: 0.9750
54	[54]	NG	Vowel	PD(30), HC(22)	RNN, CNN	RP	Detection	Accuracy: 0.7000
55	[55]	CFS	Vowel	PD(62), HC(51)	NN	BLA	Identification	Accuracy: 1.0000 Sensitivity: 1.0000, Specificity: 1.0000
56	[56]	CFS	Vowel	PD(23), HC(24)	SVM	BLA, MFCC, WT, TQWT	Diagnosis	Accuracy: 0.9311 (MALE/FEMALE Accuracy given in a graph, not readable.)
57	[57]	CFS	Vowel	PD(335),RBD(112) HC(92)	RF	BLA	PD/RBD Classification	MALE -> RBD vs PD -> Sensitivity: 0.7480, Specificity: 0.7530, RBD vs HC -> Sensitivity: 0.5980, Specificity: 0.7400, PD vs HC -> Sensitivity: 0.5530, Specificity: 0.7260, FEMALE -> RBD vs PD -> Sensitivity: 0.5190, Specificity: 0.4670, RBD vs HC -> Sensitivity: 0.4670, Specificity: 0.2630, PD vs HC -> Sensitivity: 0.4830, Specificity: 0.3680
58	[58]	Public Italian Corpus, CFS	Vowel, Syllable, Scripted Speech	PD(54), HC(40)	SVM	MFCC	Analysis	Accuracy: 0.9700, Precision: 0.9600, Recall: 1.0000, Specificity: 0.9300, F1-score: 0.9800, AUC: 0.9600
59	[59]	UCI	Vowel	PD(188), HC(64)	SVM	BLA, MFCC, WT, TQWT	Detection	Accuracy: 0.9350. F1-score: 0.9510, MCC: 0.7880
60	[60]	CFS	Vowel, syllable, Scripted speech, Picture description	PD(16)	RNN	Spectrogram	Differentiate the Dopaminergic Response	Accuracy: 0.8200, Sensitivity: 0.8600, Specificity: 0.7800, Precision: 0.80
61	[61]	UCI	Vowel	PD(20), HC(20)	SVM	BLA, MFCC	Detection	Accuracy: 0.8750, Sensitivity: 0.8500, Specificity: 0.9000, MCC: 0.7509, Precision: 0.75
62	[62]	CFS	Sentence repetition, Scripted Speech	PD(117), RBD(41), HC(98)	SVM	BLA, Verbal fluency, Rhythmic abilities	Detection	MALE -> Accuracy: 0.8900, FEMALE -> Accuracy: 0.6300

Table 8. Summary of included studies.

Nr	Ref.	Dataset	Recording	Subjects	Classifier	Feature	Aim	Performance
63	[63]	CFS	Free speech, Sentence repetition, Scripted speech, Syllable	PD(115), HC(91)	DNN	MFCC	Detection	7–15 ≺ point improvement
64	[64]	CFS	Vowel	PD(22), HC(25)	SVM	BLA	Categorization	Accuracy: 0.8680, Sensitivity: 0.9690, Specificity: 0.7900
65	[65]	UCI	Vowel	PD(188), HC(64)	XGBoost	BLA, MFC, TQWT	Detection	Accuracy: 0.9388, Sensitivity: 0.9166, Specificity: 0.9609, Precision: 0.9519, AUC: 0, 9780
66	[66]	CFS	Screpted speech	PD(61), HC(43)	NN	Spectrogram	evaluation and screening	Accuracy: 0.8964, Sensitivity: 0.9543, Specificity: 0.8440, Precision: 0.8468, F-score: 0, 8974
67	[67]	Public Italian Corpus, CFS	Vowel, syllable	PD(34), HC(41)	LR	BLA	Detection	Accuracy: 0.900, Sensitivity: 0.9690, Specificity: 0.8900
68	[68]	UCI	Vowel	PD(188), HC(64)	SVM	BLA, MFC, TQWT	Classification	Accuracy: 0.8660, Precision: 0.887, Recall: 0.9430, F1.Score: 0.914, AUC: 0.0.527
69	[69]	UCI	Vowel	PD(188), HC(64)	CNN	BLA, TQWT, MFCC, WT	Detection	F1.Score: 0.9030, AUROC: 0.8950
70	[70]	NG	Vowel	PD(188), HC(64))	KNN	TQWT	Detection	Accuracy: 0.9800, Kappa: 0.9840
71	[71]	UCI	Vowel	PD(188), HC(64)	RF	BLA, MFCC	Detection	Accuracy: 0.8884, Sensitivity: 0.9851, Specificity: 0.7008
72	[72]	CFS	Scripted speech, Numbers	Ataxic(223), Hypokinetic(423), HC(158)	CNN	Spectrogram	Differentiation	Hypokinetic dysarthria -> AUC: 0.9200, Ataxia ->AUC: 0.9300, Hypokinetic dysarthria vs Ataxia -> AUC: 0.9600
73	[73]	CLP, PC-GITA	Free speech, Syllable	PD(50), CLIP(135), HC(108)	CNN	Spectrogram	Detection	Accuracy: 0.9637
74	[74]	CFS, PC-GITA	Vowel, scripted speech	PD(80), HC(65)	CNN	Spectrogram	Detection	Accuracy: 0.816
75	[75]	UCI	Vowel	PD(188), HC(64)	NN	BLA, TQWT, MFCC, WT	Detection	Accuracy: 0.9200
76	[76]	Public Italian Corpus	Vowel, Syllable, Scripted speech	PD(44), HC(58)	CNN	Spectrogram	Diagnosis	Accuracy: 1.0000
77	[77]	UCI	Vowel	PD(188), HC(64)	SVM	BLA, TQWT, MFCC, WT	Detection	Accuracy: 0.9621
78	[78]	UCI	Vowel	PD(188), HC(64)	NN	BLA, TQWT, MFCC	Detection	Accuracy: 0.9974
79	[79]	mPower	Vowel	PD(212), HC(212)	GB	BLA, MFCC	Detection	Accuracy: 0.7143
80	[80]	UCI	Free speech	PD(42)	SVR	BLA	Progression	MAE: 3.4975, RMSE: 4.6300, R2: 0.7375
81	[81]	UCI	Free speech	PD(23), HC(8)	RNN-LSTM	BLA, MFCC	Detection	Accuracy: 0.9540, Precision: 0.9580, F1-Score: 0.9340, MCC: 0.865
82	[82]	CFS	Vowel	PD(36), HC(36)	SVM	BLA, MFCC, WT	Detectionn	Accuracy: 1.0000, Sensitivity: 1.0000, Specificity: 1.0000
83	[83]	CFS	Vowel	PD-FOG(40), PD-nFOG(40), HC(40)	SVM	BLA, MFCC, WT	Classification	Accuracy: 0.7357, Sensitivity: 0.7571, Specificity: 0.7143
84	[84]	PC-GITA, Viswanathan's dataset	Vowel	PD(64), HC(72)	SVM	BLA	Detection	Accuracy: 0.96, Sensitivity: 0.9650, Specificity: 0.9650
85	[85]	UCI	Vowel, Numbers, Scripted speech	PD(48), HC(20)	ANN	BLA	Detection	Accuracy: 0.9100, Sensitivity: 0.9900, Specificity: 0.8200
86	[86]	UCI	Vowel, Numbers, Scripted speech	PD(48), HC(20)	SVM	MFCC	Detection	Accuracy: 0.9900
87	[87]	UCI	Vowel	PD(188), HC(64)	DT	MFCC, WT, TQWT	Detection	Accuracy: 0.9680, Sensitivity: 0.9688, Specificity: 0.9688
**Alzheimer's Disease / Dementia (AD)**								
1	[88]	CFS	Conversation	AD(15), HC(15)	SVM	LP, SP, Frosodic features	Recognition	Accuracy: 0.8300, Precision: 0.9000, Recall: 0.9000, F1-score: 0.90, AUC: 0.8900
2	[89]	CFS	Scripted speech, Conversation	MD(10), HC(10)	SVM	Prosodic features	MD identification	Accuracy: 0.8500, Sensitivity: 0.8180, Specificity: 0.8880
3	[90]	CFS	Free speech	AD(26), MCI(23), HC(15)	SVM	Vocal features	Pre-dementia and AD assessment	MCI vs HC -> Equal erro rate: 21, Equal Specificity-Sensitivity: 0.7900, AD vs HC -> Equal erro rate: 13, Equal Specificity-Sensitivity: 0.8700, MCI vs AD -> Equal erro rate: 20, Equal Specificity-Sensitivity: 0.8000
4	[91]	CFS	Conversation	AD(24), HC(99)	RF,XGBoost, LR	Vocal features	Dementia risk identification	Sensitivity: 1.0000, Specificity: 1.0000
5	[92]	CFS	Questionnaire answers	AD(16), HC(17)	SVM	Speech statistic	Detetction	AUC: 0.9300
6	[93]	CFS	Conversation	AD(28), HC(51)	SVM	Linguistic features	Detetction	Accuracy: 0.8350
7	[94]	CFS	Free speech	AD(30), HC(30)	KNN	Bi spectrum	Diagnosis	Accuracy: 0.9771

Table 8. Summary of included studies.

Nr	Ref.	Dataset	Recording	Subjects	Classifier	Feature	Aim	Performance
8	[95]	CFS	Free speech	AD(25), MCI(25), HC(25)	SVM	BLA, Linguistic	Classification	Accuracy: 0.8600, Specificity: 0.7780, Precision: 0.8750, Recall: 0.9200, F1-score: 0.8600
9	[96]	ADBC	Picture description	AD(21), CE(189), MCI(19), HC(98)	MLP	lexicosyntactic features, n-gram vocabulary spaces	Prediction	Accuracy: 0.9519, F1-score: 0.9501, AUC: 0.9689
10	[97]	CFS	Free speech	Dementia(29), Depression(24)	SVM	BLA, MFCC, GTCC	Classification	Accuracy: 0.933, F1-score: 0.9350, MCC: 0.8780
11	[98]	CFS	Scripted speech	AD(16), MCI(16), HC(16)	KNN	BLA	Prediction	Accuracy: 0.8500
12	[99]	ADBC	Picture description	AD(167), HC(97)	LR	BLA, Linguistic features	Identification	Accuracy: 0.8192
13	[100]	ADBC	Picture description	AD(169), HC(99)	LR	BLA, Linguistic features	Detection	Accuracy: 0.8540
14	[101]	ADBC	Picture description	AD(137), HC(43)	CNN	Spectrogram	Clasification	Accuracy: 0.9330, F1-Score: 0.8850
15	[102]	ADBC	Picture description	AD(247), HC(178)	NN	BLA, Linguistic	Detection	AUC: 0.846
16	[103]	ADBC	Picture description	AD(139), HC(90)	NN	BLA, Spectrum, Prosodic	Assesment	AUC: 0.8600, F1-Score: 0.8400, Sensitivity: 0.8100, Specificity: 0.7700
17	[104]	ADBC	Picture description	AD(79), MCI(93) HC(108)	SVM	BLA	Clasification	Accuracy: 0.8370, Precision: 0.8380, Recall: 0.8380, F1-Score: 0.8380
18	[105]	ADBC	Picture description	AD(81), HC(76)	RF	BLA, MFCC	Screening	Accuracy: 0.8220
				**Cognitive Impairment / Mild Cognitive Impairment (CI/MCI)**				
1	[106]	CFS	Free speech	MCI(48) HC(38)	RF	Speech features	Detection	Accuracy: 0.7500, Sensitivity: 0.8130 , Specificity: 0.6670, Precision: 0.7650, F1-score: 0.7880
2	[107]	CFS	Vowel, Scripted speech	MCI(1601), GCI(367), MCI-GCI(468), HC(6343)	LR	BLA	Detection	MCI -> AUC: 0.6128, GCI -> AUC: 0.6731, MCI-GCI -> AUC: 0.7712
3	[108]	CFS	Free sppech	Apathy(30) non-Apathy(30)	LR	BLA	Detection	MALE -> AUC: 0.8800, FEMALE -> AUC: 0.7700
4	[109]	CFS	Free speech	MCI(16), HC(16)	SVM	Linguistic features	Varbal fluency analysis	AD -> AUC: 0.9390, MCI -> AUC: 0.7580
5	[110]	NG	Scripted speech	AD(24), MCI(47), MD(38)	DNN	BLA	Detection	Accuracy: 0.8400
6	[111]	CFS	Picture description	MCI(25), HC(30)	RF	BLA, Linguistic features, Language features	Identification	Accuracy: 0.9600, Precision: 0.9600, Recall: 0.9600, F1-score: 0.9600
7	[112]	CFS	Picture description	AD(27), MCI(44), SCI(16), MD(38)	SVM	Vocal features1	Classification	SCI vs AD -> Accuracy:0.9200, SCI vs MD -> Accuracy:0.9200, SCI vs MCI -> Accuracy:0.8600, MCI vs AD -> Accuracy:0.8600, MCI vs MD -> Accuracy:0.8200, SCI vs MCI, AD Accuracy:0.7800, SCI vs MCI, Mixed -> Accuracy:0.7400, SCI vs MCI and all others -> Accuracy:0.7500
8	[113]	CFS	Picture description	MCI(48) HC(48)	RNN	Spectrogram	Detection	Accuracy: 0.9057, Precision: 0.9084, Recall: 0.9056, F1-Score: 0.9070
				**Covid 19**				
1	[114]	Coswara database	Vowel	Covid-19(83), HC(83)	RF	BLA, MFCC	Detection	Accuracy: 0.8529, Sensitivity: 0.7647, Specificity: 0.9412, Precision: 0.9286, AUC: 0.8670, F1-score: 0.8387
2	[115]	Coswara database	Vowel	Covid-19(77), HC(950)	SVM	BLA, MFCC	Detection	Accuracy: 0.9700
3	[116]	Coswara database	Scripted speech	Covid-19(308), HC(585)	KNN	Spectrogram	Analysis	Accuracy: 0.7970, AUC: 0.8300
4	[117]	Coswara database	Vowel, Numbers	NG	NN	BLA, MFCC	Detection	Accuracy: 0.9176, Sensitivity: 0.9218, Specificity: 0.9219
5	[118]	CFS	Scripted speech	Covid-19(106), HC(106)	CNN	Spectrogram	Detection	AUROC: 0.7900, Sensitivity: 0.7500, Specificity: 0.71
6	[119]	Coswara database, Multiple datasets	Vowel, Scripted speech, Numbers	Covid-19(387), HC(506)	LGM	Spectrogram	Detection	Accuracy: 0.9700, AUC: 0.9900
7	[120]	CHESS	Vowel, Numbers	Covid-19(127), HC(127)	ELM	MLA, MFCC	Detection	Accuracy: 0.9615, Precision: 0.9286, Recall: 1.0000, F1-Score: 0.9630
				**Amyotrophic lateral sclerosis (ALS)**				
1	[121]	CFS	Vowel, Scripted speech	ALS(13), HC(10)	RF	BLA	Assessment	Accuracy: 0.9697
2	[122]	CFS	Scripted speech	ALS(12), HC(2)	SVM	BLA, Articulatory features	Prediction of intelligibility	R2: 0.712, RMSE:37.5620

Table 8. Summary of included studies.

Nr	Ref.	Dataset	Recording	Subjects	Classifier	Feature	Aim	Performance
3	[123]	CFS	Vowel	ALS+Bulbar(14), ALS-Bulbar(32), HC(18)	SVM	BLA	Bulbar involvement detection	Accuracy: 0.9580, Sensitivity: 0.9140 , Specificity: 0.9930
4	[124]	CFS	Vowel	ALS(58), HC(6)	NG	BLA	Vital capacity estimation	MAE: 0.1850
				**Depression**				
1	[125]	DAIC-WOZ, RAVDESS, AVi-D databases	Interviews	Depression(54), HC(127)	RNN	BLA, MFCC. Linguistic	Recognition	Accuracy: 0.7627, RMSE: 0.4000
2	[126]	Chines databases	Interviews	Mixed(1698)	LSTM	MFCC	Detection	AUC: 0.8682, Sensitivity: 0.8214, Specificity: 0.8035
3	[127]	DAIC-WOZ	Interviews	Depression(70), HC(29)	CNN	Spectrogram	Prediction	Accuracy: 0.7740, F1-Score: 0.8230
4	[128]	CFS	Interviews	Depression(73), HC(47)	SVM	BLA, Spectrogram	Detection	Accuracy: 0.7400
				**Cardiovascular disease (CD)**				
1	[129]	CFS	Vowel	CD(35), HC(40)	KNN	BLA	Classification	Accuracy: 0.8151, Sensitivity: 0.8246, Specificity: 0.8056
2	[130]	CFS	Vowel, Number 33	CD(58)	KNN	MFCC	Predicting congestive heart failure	Accuracy: 0.945
				**Essential tremor (ET)**				
1	[131]	CFS	Vowel	ET(20), HC(20)	Radial-k SVM	Mean Logarithm of variance and range	Classification	Sensitivity: 1.0000, Specificity: 0.9128, F1-score: 0.9665
2	[132]	CFS	Vowel	ET(58), HC(74)	SVM	Spectrogram	Diagnosis	Accuracy: 0.9830, Sensitivity: 0.9830, Specificity: 0.9830, PPV: 0.9830, NPV: 0.9830, AUC: 0.9940
				**Multipel skleros (MS)**				
1	[133]	CFS	Syllable	MS(120), HC(60)	CNN	Spectrogram	Daidochokinesis assessment	Accuracy: 0.9890
2	[134]	CFS	Picture description, Free speech	MS(65), HC(66)	RF	BLA	Speech analysis	Accuracy: 0.8200, AUC: 0.7600
				**Stroke**				
1	[135]	CFS	Vowel, Syllable	Stroke(104), HC(78)	LSTM	MFCC	Recognition	Accuracy: 0.8480
2	[136]	CFS	Picture description, Scripted speech	Stroke159, CD(62)	NN	Spectrogram	Screening	Accuracy: 0.7240, Sensitivity: 0.3226, Specificity: 0.8805, AUC: 0.7163
				**Autism**				
1	[137]	CFS	Free speech	ASD(20), HC(38)	SNN	Spectrogram	Classification	Accuracy: 0.7900, Precision: 0.8040, Recall: 0.7930, AUROC: 0.8220, F1-score: 0.7900
				**Fatigue**				
1	[138]	CFS	Vowel, Scripted text	F(15)	SVM	BLA, MFCC	Fatigue Detection	Accuracy: 0.8100
				**Neurodegenerative cognitive complaints (NCC)**				
1	[139]	CFS	Questionnaire recordings	FMD(15), ND(15)	SVM	Speech and silent, quality and spectral features	FMD,ND Identification	Accuracy: 0.9620
				**Functional dysphagia(FD),Oropharyngeal dysphagia (OD)**				
1	[140]	CFS	Vowel, Syllable, Free speech	FD/OD(46), HC(46)	RF	BLA, MFCC	FD/OD analysis	Accuracy: 0.8500, Sensitivity: 0.9100, Precision: 0.8400, AUC: 0.8600, F1-score: 0.8600
				**Traumatic Brain Injury (TBI)**				
1	[141]	Coelho corpus	Free speech	TBI(55), HC(52)	CNN	Spectrogram	Detection	Accuracy: 0.8387
				**Asthma**				
1	[142]	CFS	Free speech	Asthma(26), HC(52)	RF	BLA	Asthma pulmonary capacity prediction	Accuracy: 0.8000, Sensitivity: 0.4000, Precision: 0.8000, AUC: 0.8400, F1-score: 0.7900
				**Chronic Obstructive Pulmonary Disease (COPD)**				
1	[143]	CFS	Scripted speech	COPD(40), HC(19)	RF	BLA	COPD supervision	Accuracy: 0.7500, Sensitivity: 0.8110, Specificity: 0.7100
				**Influenza disease**				
1	[144]	CFS	Vowel	ID(16), HC(16)	KNN	DWT	Classification	Accuracy: 0.9170, AUC: 0.9600
				**Neurological disease (ND)**				

Table 8. Summary of included studies.

Nr	Ref.	Dataset	Recording	Subjects	Classifier	Feature	Aim	Performance
1	[145]	NG	Vowel	PD(30), MSA(9), FND(5), Somatization(1), Dysthonia(1), CD(2), ET(1), GPD(1)	KNN	BLA, MFCC	Detection	Accuracy: 0.8800, Sensitivity: 0.9000, Specificity: 0.8500, MCC: 0.7500

References

1. Sakar CO, Serbes G, Gunduz A, Tunc HC, Nizam H, Sakar BE, et al. A comparative analysis of speech signal processing algorithms for Parkinson's disease classification and the use of the tunable Q-factor wavelet transform. Applied Soft Computing. 2019;74:255–263. doi:10.1016/j.asoc.2018.10.022.

2. Moro-Velazquez L, Gomez-Garcia JA, Godino-Llorente JI, Villalba J, Rusz J, Shattuck-Hufnagel S, et al. A forced gaussians based methodology for the differential evaluation of Parkinson's Disease by means of speech processing. Biomedical Signal Processing and Control. 2019;48:205–220. doi:10.1016/j.bspc.2018.10.020.

3. Meghraoui D, Boudraa B, Merazi-Meksen T, Gómez Vilda P. A novel pre-processing technique in pathologic voice detection: Application to Parkinson's disease phonation. Biomedical Signal Processing and Control. 2021;68:102604. doi:10.1016/j.bspc.2021.102604.

4. Quan C, Ren K, Luo Z. A Deep Learning Based Method for Parkinson's Disease Detection Using Dynamic Features of Speech. IEEE access : practical innovations, open solutions. 2021;9:10239–10252. doi:10.1109/ACCESS.2021.3051432.

5. Goyal J, Khandnor P, Aseri TC. A Hybrid Approach for Parkinson's Disease diagnosis with Resonance and Time-Frequency based features from Speech signals. Expert Systems with Applications. 2021;182:115283. doi:10.1016/j.eswa.2021.115283.

6. Lamba R, Gulati T, Alharbi HF, Jain A. A hybrid system for Parkinson's disease diagnosis using machine learning techniques. International Journal of Speech Technology. 2021;doi:10.1007/s10772-021-09837-9.

7. Nilashi M, Ibrahim O, Ahmadi H, Shahmoradi L, Farahmand M. A hybrid intelligent system for the prediction of Parkinson's Disease progression using machine learning techniques. Biocybernetics and Biomedical Engineering. 2018;38(1):1–15. doi:10.1016/j.bbe.2017.09.002.

8. Cantürk I, Karabiber F. A Machine Learning System for the Diagnosis of Parkinson's Disease from Speech Signals and Its Application to Multiple Speech Signal Types. Arabian Journal for Science and Engineering. 2016;41(12):5049–5059. doi:10.1007/s13369-016-2206-3.

9. Carrón J, Campos-Roca Y, Madruga M, Pérez CJ. A mobile-assisted voice condition analysis system for Parkinson's disease: assessment of usability conditions. Biomedical Engineering Online. 2021;20(1):114. doi:10.1186/s12938-021-00951-y.

10. Yücelbaş C. A new approach: information gain algorithm-based k-nearest neighbors hybrid diagnostic system for Parkinson's disease. Physical and Engineering Sciences in Medicine. 2021;44(2):511–524. doi:10.1007/s13246-021-01001-6.

11. Cai Z, Gu J, Chen HL. A New Hybrid Intelligent Framework for Predicting Parkinson's Disease. IEEE access : practical innovations, open solutions. 2017;5:17188–17200. doi:10.1109/ACCESS.2017.2741521.

12. Tuncer T, Dogan S. A novel octopus based Parkinson's disease and gender recognition method using vowels. Applied Acoustics. 2019;155:75–83. doi:10.1016/j.apacoust.2019.05.019.

13. Jahnavi BS, Supraja BS, Lalitha S. A vital neurodegenerative disorder detection using speech cues. Journal of Intelligent & Fuzzy Systems. 2020;38(5):6337–6345. doi:10.3233/JIFS-179714.

14. Gunduz H. An efficient dimensionality reduction method using filter-based feature selection and variational autoencoders on Parkinson's disease classification. Biomedical Signal Processing and Control. 2021;66:102452. doi:10.1016/j.bspc.2021.102452.

15. Zhang L, Qu Y, Jin B, Jing L, Gao Z, Liang Z. An Intelligent Mobile-Enabled System for Diagnosing Parkinson Disease: Development and Validation of a Speech Impairment Detection System. JMIR medical informatics. 2020;8(9):e18689. doi:10.2196/18689.

16. Cai Z, Gu J, Wen C, Zhao D, Huang C, Huang H, et al. An Intelligent Parkinson's Disease Diagnostic System Based on a Chaotic Bacterial Foraging Optimization Enhanced Fuzzy KNN Approach. Computational and Mathematical Methods in Medicine. 2018;2018:2396952. doi:10.1155/2018/2396952.

17. Rizvi D, Nissar I, Masood S, Ahmed M, Ahmad F. An LSTM based Deep learning model for voice-based detection of Parkinson's disease. 2020;29:337–343.

18. Olivares R, Munoz R, Soto R, Crawford B, Cárdenas D, Ponce A, et al. An optimized brain-based algorithm for classifying parkinson's disease. Applied Sciences (Switzerland). 2020;10(5). doi:10.3390/app10051827.

19. Tougui I, Jilbab A, Mhamdi JE. Analysis of Smartphone Recordings in Time, Frequency, and Cepstral Domains to Classify Parkinson's Disease. Healthcare Informatics Research. 2020;26(4):274–283. doi:10.4258/hir.2020.26.4.274.

20. Solana-Lavalle G, Rosas-Romero R. Analysis of voice as an assisting tool for detection of Parkinson's disease and its subsequent clinical interpretation. Biomedical Signal Processing and Control. 2021;66:102415. doi:10.1016/j.bspc.2021.102415.

21. Pramanik M, Pradhan R, Nandy P, Qaisar SM, Bhoi AK. Assessment of Acoustic Features and Machine Learning for Parkinson's Detection. Journal of Healthcare Engineering. 2021;2021:1–13. doi:10.1155/2021/9957132.

22. Ali L, Zhu C, Zhang Z, Liu Y. Automated Detection of Parkinson's Disease Based on Multiple Types of Sustained Phonations Using Linear Discriminant Analysis and Genetically Optimized Neural Network. IEEE journal of translational engineering in health and medicine. 2019;7:2000410. doi:10.1109/JTEHM.2019.2940900.

23. Braga D, Madureira AM, Coelho L, Ajith R. Automatic detection of Parkinson's disease based on acoustic analysis of speech. Engineering Applications of Artificial Intelligence. 2019;77:148–158. doi:10.1016/j.engappai.2018.09.018.

24. Novotný M, Rusz J, Čmejla R, Růžička E. Automatic evaluation of articulatory disorders in Parkinson's disease. IEEE Transactions on Audio, Speech and Language Processing. 2014;22(9):1366–1378. doi:10.1109/TASLP.2014.2329734.

25. Solana-Lavalle G, Galán-Hernández JC, Rosas-Romero R. Automatic Parkinson disease detection at early stages as a pre-diagnosis tool by using classifiers and a small set of vocal features. Biocybernetics and Biomedical Engineering. 2020;40(1):505–516. doi:10.1016/j.bbe.2020.01.003.

26. Zhang HH, Yang L, Liu Y, Wang P, Yin J, Li Y, et al. Classification of Parkinson's disease utilizing multi-edit nearest-neighbor and ensemble learning algorithms with speech samples. BioMedical Engineering Online. 2016;15(1). doi:10.1186/s12938-016-0242-6.

27. Khan T, Westin J, Dougherty M. Classification of speech intelligibility in Parkinson's disease. Biocybernetics and Biomedical Engineering. 2014;34(1):35–45. doi:10.1016/j.bbe.2013.10.003.

28. Berus L, Klancnik S, Brezocnik M, Ficko M. Classifying parkinson's disease based on acoustic measures using artificial neural networks. Sensors (Switzerland). 2019;19(1). doi:10.3390/s19010016.

29. García AM, Arias-Vergara T, C Vasquez-Correa J, Nöth E, Schuster M, Welch AE, et al. Cognitive Determinants of Dysarthria in Parkinson's Disease: An Automated Machine Learning Approach. Movement Disorders: Official Journal of the Movement Disorder Society. 2021;36(12):2862–2873. doi:10.1002/mds.28751.

30. Sakar BE, Isenkul ME, Sakar CO, Sertbas A, Gurgen F, Delil S, et al. Collection and Analysis of a Parkinson Speech Dataset With Multiple Types of Sound Recordings. IEEE Journal of Biomedical and Health Informatics. 2013;17(4):828–834. doi:10.1109/JBHI.2013.2245674.

31. Viswanathan R, Arjunan SP, Bingham A, Jelfs B, Kempster P, Raghav S, et al. Complexity measures of voice recordings as a discriminative tool for Parkinson's disease. Biosensors. 2020;10(1). doi:10.3390/bios10010001.

32. Hireš M, Gazda M, Drotár P, Pah ND, Motin MA, Kumar DK. Convolutional neural network ensemble for Parkinson's disease detection from voice recordings. Computers in Biology and Medicine. 2022;141:105021. doi:10.1016/j.compbiomed.2021.105021.

33. MAJDA-ZDANCEWICZ E, POTULSKA-CHROMIK A, JAKUBOWSKI J, NOJSZEWSKA M, KOSTERA-PRUSZCZYK A. Deep learning vs feature engineering in the assessment of voice signals for diagnosis in Parkinson's disease. Bulletin of the Polish Academy of Sciences: Technical Sciences. 2021;69(3). doi:10.24425/bpasts.2021.137347.

34. Ozkanca Y, Göksu Öztürk M, Ekmekci MN, Atkins DC, Demiroglu C, Hosseini Ghomi R. Depression Screening from Voice Samples of Patients Affected by Parkinson's Disease. Digital Biomarkers. 2019;3(2):72–82. doi:10.1159/000500354.

35. Rahman W, Lee S, Islam MS, Antony VN, Ratnu H, Ali MR, et al. Detecting Parkinson Disease Using a Web-Based Speech Task: Observational Study. Journal of Medical Internet Research. 2021;23(10):e26305. doi:10.2196/26305.

36. Almeida JS, Rebouças Filho PP, Carneiro T, Wei W, Damaševičius R, Maskeliūnas R, et al. Detecting Parkinson's disease with sustained phonation and speech signals using machine learning techniques. Pattern Recognition Letters. 2019;125:55–62. doi:10.1016/j.patrec.2019.04.005.

37. Benba A, Jilbab A, Hammouch A. Detecting multiple system atrophy, Parkinson and other neurological disorders using voice analysis. International Journal of Speech Technology. 2017;20(2):281–288. doi:10.1007/s10772-017-9404-6.

38. Arora S, Baghai-Ravary L, Tsanas A. Developing a large scale population screening tool for the assessment of Parkinson's disease using telephone-quality voice. Journal of the Acoustical Society of America. 2019;145(5):2871–2884. doi:10.1121/1.5100272.

39. Yang S, Zheng F, Luo X, Cai S, Wu Y, Liu K, et al. Effective Dysphonia Detection Using Feature Dimension Reduction and Kernel Density Estimation for Patients with Parkinson's Disease. PLoS ONE. 2014;9(2):e88825. doi:10.1371/journal.pone.0088825.

40. Oung QW, Muthusamy H, Basah SN, Lee H, Vijean V. Empirical Wavelet Transform Based Features for Classification of Parkinson's Disease Severity. Journal of Medical Systems. 2018;42(2). doi:10.1007/s10916-017-0877-2.

41. Haq AU, Li JP, Memon MH, khan J, Malik A, Ahmad T, et al. Feature Selection Based on L1-Norm Support Vector Machine and Effective Recognition System for Parkinson's Disease Using Voice Recordings. IEEE access : practical innovations, open solutions. 2019;7:37718–37734. doi:10.1109/ACCESS.2019.2906350.

42. Rusz J, Novotný M, Hlavnička J, Tykalová T, Růžička E. High-Accuracy Voice-Based Classification Between Patients With Parkinson's Disease and Other Neurological Diseases May Be an Easy Task With Inappropriate Experimental Design. IEEE Transactions on Neural Systems and Rehabilitation Engineering. 2017;25(8):1319–1321. doi:10.1109/TNSRE.2016.2621885.

43. Tracy JM, Özkanca Y, Atkins DC, Hosseini Ghomi R. Investigating voice as a biomarker: Deep phenotyping methods for early detection of Parkinson's disease. Journal of Biomedical Informatics. 2020;104:103362. doi:10.1016/j.jbi.2019.103362.

44. Klempíř O, Krupička R. MACHINE LEARNING USING SPEECH UTTERANCES FOR PARKINSON DISEASE DETECTION. Lékař a technika - Clinician and Technology. 2018;48(2):66–71.

45. Alhussein M. Monitoring Parkinson's Disease in Smart Cities. IEEE access : practical innovations, open solutions. 2017;5:19835–19841. doi:10.1109/ACCESS.2017.2748561.

46. Tsanas A, Little MA, McSharry PE, Spielman J, Ramig LO. Novel speech signal processing algorithms for high-accuracy classification of Parkinson's disease. IEEE transactions on bio-medical engineering. 2012;59(5):1264–1271. doi:10.1109/TBME.2012.2183367.

47. Tsanas A, Little MA, Fox C, Ramig LO. Objective automatic assessment of rehabilitative speech treatment in Parkinson's disease. IEEE Transactions on Neural Systems and Rehabilitation Engineering. 2014;22(1):181–190. doi:10.1109/TNSRE.2013.2293575.

48. Gómez-Vilda P, Mekyska J, Ferrández JM, Palacios-Alonso D, Gómez-Rodellar A, Rodellar-Biarge V, et al. Parkinson disease detection from speech articulation neuromechanics. Frontiers in Neuroinformatics. 2017;11. doi:10.3389/fninf.2017.00056.

49. Zhang T, Zhang Y, Sun H, Shan H. Parkinson disease detection using energy direction features based on EMD from voice signal. Biocybernetics and Biomedical Engineering. 2021;41(1):127–141. doi:10.1016/j.bbe.2020.12.009.

50. Karan B, Sahu SS, Mahto K. Parkinson disease prediction using intrinsic mode function based features from speech signal. Biocybernetics and Biomedical Engineering. 2020;40(1):249–264. doi:10.1016/j.bbe.2019.05.005.

51. Laganas C, Iakovakis D, Hadjidimitriou SK, Charisis V, Dias SB, Bostanjopoulou S, et al. Parkinson's Disease Detection Based on Running Speech Data From Phone Calls. IEEE Transactions on Biomedical Engineering. 2021;doi:10.1109/TBME.2021.3116935.

52. Bchir O. Parkinson's Disease Classification using Gaussian Mixture Models with Relevance Feature Weights on Vocal Feature Sets. International Journal of Advanced Computer Science and Applications. 2020;11(4). doi:10.14569/IJACSA.2020.0110456.

53. Rahman A, Rizvi SS, Khan A, Abbasi AA, Khan SU, Chung TS. Parkinson's disease diagnosis in cepstral domain using MFCC and dimensionality reduction with SVM classifier. Mobile Information Systems. 2021;2021. doi:10.1155/2021/8822069.

54. Fujita T, Luo Z, Quan C, Mori K, Cao S. Performance evaluation of rnn with hyperbolic secant in gate structure through application of parkinson's disease detection. Applied Sciences (Switzerland). 2021;11(10). doi:10.3390/app11104361.

55. Vital TPR, Nayak J, Naik B, Jayaram D. Probabilistic Neural Network-based Model for Identification of Parkinson's Disease by using Voice Profile and Personal Data. Arabian Journal for Science and Engineering. 2021;46(4):3383–3407. doi:10.1007/s13369-020-05080-7.

56. Azadi H, Akbarzadeh-T MR, Kobravi HR, Shoeibi A. Robust Voice Feature Selection Using Interval Type-2 Fuzzy AHP for Automated Diagnosis of Parkinson's Disease. IEEE/ACM Transactions on Audio, Speech, and Language Processing. 2021;29:2792–2802. doi:10.1109/TASLP.2021.3097215.

57. Arora S, Lo C, Hu M, Tsanas A. Smartphone Speech Testing for Symptom Assessment in Rapid Eye Movement Sleep Behavior Disorder and Parkinson's Disease. IEEE access : practical innovations, open solutions. 2021;9:44813–44824. doi:10.1109/ACCESS.2021.3057715.

58. Amato F, Borzì L, Olmo G, Artusi CA, Imbalzano G, Lopiano L. Speech Impairment in Parkinson's Disease: Acoustic Analysis of Unvoiced Consonants in Italian Native Speakers. IEEE access : practical innovations, open solutions. 2021;9:166370–166381. doi:10.1109/ACCESS.2021.3135626.

59. Hoq M, Uddin MN, Park SB. Vocal Feature Extraction-Based Artificial Intelligent Model for Parkinson's Disease Detection. Diagnostics (Basel, Switzerland). 2021;11(6):1076. doi:10.3390/diagnostics11061076.

60. Jain A, Abedinpour K, Polat O, Çalışkan MM, Asaei A, Pfister FMJ, et al. Voice Analysis to Differentiate the Dopaminergic Response in People With Parkinson's Disease. Frontiers in Human Neuroscience. 2021;15:667997. doi:10.3389/fnhum.2021.667997.

61. Benba A, Jilbab A, Hammouch A. Voice assessments for detecting patients with Parkinson's diseases using PCA and NPCA. International Journal of Speech Technology. 2016;19(4):743–754. doi:10.1007/s10772-016-9367-z.

62. Jeancolas L, Mangone G, Petrovska-Delacrétaz D, Benali H, Benkelfat BE, Arnulf I, et al. Voice characteristics from isolated rapid eye movement sleep behavior disorder to early Parkinson's disease. Parkinsonism and Related Disorders. 2022;95:86–91. doi:10.1016/j.parkreldis.2022.01.003.

63. Jeancolas L, Petrovska-Delacrétaz D, Mangone G, Benkelfat BE, Corvol JC, Vidailhet M, et al. X-Vectors: New Quantitative Biomarkers for Early Parkinson's Disease Detection From Speech. Frontiers in Neuroinformatics. 2021;15. doi:10.3389/fninf.2021.578369.

64. Saloni S, Sharma R, Gupta A. Human Voice Waveform Analysis for Categorization of Healthy and Parkinson Subjects. International Journal of Healthcare Information Systems and Informatics. 2016;11:21–35. doi:10.4018/IJHISI.2016010102.

65. Lamba R, Gulati T, Jain A. A Hybrid Feature Selection Approach for Parkinson's Detection Based on Mutual Information Gain and Recursive Feature Elimination. Arabian Journal for Science and Engineering. 2022;47(8):10263–10276. doi:10.1007/s13369-021-06544-0.

66. Maskeliūnas R, Damaševičius R, Kulikajevas A, Padervinskis E, Pribuišis K, Uloza V. A Hybrid U-Lossian Deep Learning Network for Screening and Evaluating Parkinson's Disease. Applied Sciences. 2022;12(22):11601. doi:10.3390/app122211601.

67. Lamba R, Gulati T, Jain A, Rani P. A Speech-Based Hybrid Decision Support System for Early Detection of Parkinson's Disease. Arabian Journal for Science and Engineering;doi:10.1007/s13369-022-07249-8.

68. Dao SVT, Yu Z, Tran LV, Phan PNK, Huynh TTM, Le TM. An Analysis of Vocal Features for Parkinson's Disease Classification Using Evolutionary Algorithms. Diagnostics (Basel, Switzerland). 2022;12(8):1980. doi:10.3390/diagnostics12081980.

69. Barukab O, Ahmad A, Khan T, Thayyil Kunhumuhammed MR. Analysis of Parkinson's Disease Using an Imbalanced-Speech Dataset by Employing Decision Tree Ensemble Methods. Diagnostics (Basel, Switzerland). 2022;12(12):3000. doi:10.3390/diagnostics12123000.

70. Kaya D. Automated gender-Parkinson's disease detection at the same time via a hybrid deep model using human voice. Concurrency and Computation: Practice and Experience. 2022;34(26):e7289. doi:10.1002/cpe.7289.

71. Hawi S, Alhozami J, AlQahtani R, AlSafran D, Alqarni M, El Sahmarany L. Automatic Parkinson's disease detection based on the combination of long-term acoustic features and Mel frequency cepstral coefficients (MFCC). Biomedical Signal Processing and Control. 2022;78:104013. doi:10.1016/j.bspc.2022.104013.

72. Song J, Lee JH, Choi J, Suh MK, Chung MJ, Kim YH, et al. Detection and differentiation of ataxic and hypokinetic dysarthria in cerebellar ataxia and parkinsonian disorders via wave splitting and integrating neural networks. PloS One. 2022;17(6):e0268337. doi:10.1371/journal.pone.0268337.

73. Chen F, Yang C, Khishe M. Diagnose Parkinson's disease and cleft lip and palate using deep convolutional neural networks evolved by IP-based chimp optimization algorithm. Biomedical Signal Processing and Control. 2022;77. doi:10.1016/j.bspc.2022.103688.

74. Quan C, Ren K, Luo Z, Chen Z, Ling Y. End-to-end deep learning approach for Parkinson's disease detection from speech signals. Biocybernetics and Biomedical Engineering. 2022;42(2):556–574. doi:10.1016/j.bbe.2022.04.002.

75. El-Habbak MO, Abdelalim MA, Mohamed HN, Abd-Elaty MH, Hammouda AM, Mohamed YY, et al. Enhancing Parkinson's Disease Diagnosis Accuracy Through Speech Signal Algorithm Modeling. Cmc-Computers Materials & Continua. 2022;70(2):2953–2969. doi:10.32604/cmc.2022.020109.

76. Xie JC, Gan Y, Liang P, Lan R, Gao H. Exploring robust computer-aided diagnosis of Parkinson's disease based on various voice signals. Frontiers in Physics. 2022;10:1048833. doi:10.3389/fphy.2022.1048833.

77. Gafoor SHA, Theagarajan P. Intelligent approach of score-based artificial fish swarm algorithm (SAFSA) for Parkinson's disease diagnosis. International Journal of Intelligent Computing and Cybernetics. 2022;15(4):540–561. doi:10.1108/IJICC-10-2021-0226.

78. Senturk ZK. Layer recurrent neural network-based diagnosis of Parkinson's disease using voice features. Biomedizinische Technik Biomedical Engineering. 2022;67(4):249–266. doi:10.1515/bmt-2022-0022.

79. Tougui I, Jilbab A, Mhamdi JE. Machine Learning Smart System for Parkinson Disease Classification Using the Voice as a Biomarker. Healthcare Informatics Research. 2022;28(3):210–221. doi:10.4258/hir.2022.28.3.210.

80. Bárcenas R, Fuentes-García R, Naranjo L. Mixed kernel SVR addressing Parkinson's progression from voice features. PLoS ONE. 2022;17(10 October). doi:10.1371/journal.pone.0275721.

81. Almasoud AS, Eisa TAE, Al-Wesabi FN, Elsafi A, Al Duhayyim M, Yaseen I, et al. Parkinson's Detection Using RNN-Graph-LSTM with Optimization Based on Speech Signals. Cmc-Computers Materials & Continua. 2022;72(1):871–886. doi:10.32604/cmc.2022.024596.

82. Yu Q, Zou X, Quan F, Dong Z, Yin H, Liu J, et al. Parkinson's disease patients with freezing of gait have more severe voice impairment than non-freezers during "ON state". Journal of Neural Transmission. 2022;129(3):277–286. doi:10.1007/s00702-021-02458-1.

83. Motin MA, Pah ND, Raghav S, Kumar DK. Parkinson's Disease Detection Using Smartphone Recorded Phonemes in Real World Conditions. Ieee Access. 2022;10:97600–97609. doi:10.1109/ACCESS.2022.3203973.

84. Pah ND, Motin MA, Kumar DK. Phonemes based detection of parkinson's disease for telehealth applications. Scientific Reports. 2022;12(1). doi:10.1038/s41598-022-13865-z.

85. Liu W, Liu J, Peng T, Wang G, Balas VE, Geman O, et al. Prediction of Parkinson's disease based on artificial neural networks using speech datasets. Journal of Ambient Intelligence and Humanized Computing. 2022;doi:10.1007/s12652-022-03825-w.

86. Khaskhoussy R, Ben Ayed Y. Speech processing for early Parkinson's disease diagnosis: machine learning and deep learning-based approach. Social Network Analysis and Mining. 2022;12(1):73. doi:10.1007/s13278-022-00905-9.

87. Pramanik M, Pradhan R, Nandy P, Bhoi AK, Barsocchi P. The ForEx plus plus based decision tree ensemble approach for robust detection of Parkinson's disease. Journal of Ambient Intelligence and Humanized Computing;doi:10.1007/s12652-022-03719-x.

88. Nasreen S, Rohanian M, Hough J, Purver M. Alzheimer's Dementia Recognition From Spontaneous Speech Using Disfluency and Interactional Features. Frontiers in Computer Science. 2021;3. doi:10.3389/fcomp.2021.640669.

89. Gonzalez-Moreira E, Torres-Boza D, Kairuz HA, Ferrer C, Garcia-Zamora M, Espinoza-Cuadros F, et al. Automatic prosodic analysis to identify mild dementia. BioMed Research International. 2015;2015. doi:10.1155/2015/916356.

90. König A, Satt A, Sorin A, Hoory R, Toledo-Ronen O, Derreumaux A, et al. Automatic speech analysis for the assessment of patients with predementia and Alzheimer's disease. Alzheimer's & Dementia (Amsterdam, Netherlands). 2015;1(1):112–124. doi:10.1016/j.dadm.2014.11.012.

91. Shimoda A, Li Y, Hayashi H, Kondo N. Dementia risks identified by vocal features via telephone conversations: A novel machine learning prediction model. PloS One. 2021;16(7):e0253988. doi:10.1371/journal.pone.0253988.

92. Tanaka H, Adachi H, Ukita N, Ikeda M, Kazui H, Kudo T, et al. Detecting Dementia Through Interactive Computer Avatars. IEEE journal of translational engineering in health and medicine. 2017;5:2200111. doi:10.1109/JTEHM.2017.2752152.

93. Khodabakhsh A, Yesil F, Guner E, Demiroglu C. Evaluation of linguistic and prosodic features for detection of Alzheimer's disease in Turkish conversational speech. EURASIP Journal on Audio, Speech, and Music Processing. 2015;2015(1):9. doi:10.1186/s13636-015-0052-y.

94. Nasrolahzadeh M, Mohammadpoory Z, Haddadnia J. Higher-order spectral analysis of spontaneous speech signals in Alzheimer's disease. Cognitive Neurodynamics. 2018;12(6):583–596. doi:10.1007/s11571-018-9499-8.

95. Gosztolya G, Vincze V, Tóth L, Pákáski M, Kálmán J, Hoffmann I. Identifying Mild Cognitive Impairment and mild Alzheimer's disease based on spontaneous speech using ASR and linguistic features. Computer Speech and Language. 2019;53:181–197. doi:10.1016/j.csl.2018.07.007.

96. Alkenani AH, Li Y, Xu Y, Zhang Q. Predicting Alzheimer's Disease from Spoken and Written Language Using Fusion-Based Stacked Generalization. Journal of Biomedical Informatics. 2021;118:103803. doi:10.1016/j.jbi.2021.103803.

97. Sumali B, Mitsukura Y, Liang KC, Yoshimura M, Kitazawa M, Takamiya A, et al. Speech Quality Feature Analysis for Classification of Depression and Dementia Patients. Sensors (Basel, Switzerland). 2020;20(12):3599. doi:10.3390/s20123599.

98. Mirzaei S, El Yacoubi M, Garcia-Salicetti S, Boudy J, Kahindo C, Cristancho-Lacroix V, et al. Two-Stage Feature Selection of Voice Parameters for Early Alzheimer's Disease Prediction. IRBM. 2018;39(6):430–435. doi:10.1016/j.irbm.2018.10.016.

99. Fraser KC, Meltzer JA, Rudzicz F. Linguistic features identify Alzheimer's disease in narrative speech. Journal of Alzheimer's Disease. 2015;49(2):407–422. doi:10.3233/JAD-150520.

100. Guo Z, Ling Z, Li Y. Detecting Alzheimer's Disease from Continuous Speech Using Language Models. Journal of Alzheimer's disease: JAD. 2019;70(4):1163–1174. doi:10.3233/JAD-190452.

101. Bertini F, Allevi D, Lutero G, Calzà L, Montesi D. An automatic Alzheimer's disease classifier based on spontaneous spoken English. Computer Speech and Language. 2022;72. doi:10.1016/j.csl.2021.101298.

102. Agbavor F, Liang H. Artificial Intelligence-Enabled End-To-End Detection and Assessment of Alzheimer's Disease Using Voice. Brain Sciences. 2022;13(1):28. doi:10.3390/brainsci13010028.

103. Pérez-Toro PA, Rodríguez-Salas D, Arias-Vergara T, Klumpp P, Schuster M, Nöth E, et al. Interpreting acoustic features for the assessment of Alzheimer's disease using ForestNet. Smart Health. 2022;26. doi:10.1016/j.smhl.2022.100347.

104. Ying Y, Yang T, Zhou H. Multimodal fusion for alzheimer's disease recognition. Applied Intelligence;doi:10.1007/s10489-022-04255-z.

105. Hason L, Krishnan S. Spontaneous speech feature analysis for alzheimer's disease screening using a random forest classifier. Frontiers in Digital Health. 2022;4:901419. doi:10.3389/fdgth.2022.901419.

106. Toth L, Hoffmann I, Gosztolya G, Vincze V, Szatloczki G, Banreti Z, et al. A Speech Recognition-based Solution for the Automatic Detection of Mild Cognitive Impairment from Spontaneous Speech. Current Alzheimer Research. 2018;15(2):130–138. doi:10.2174/1567205014666171121114930.

107. Nagumo R, Zhang Y, Ogawa Y, Hosokawa M, Abe K, Ukeda T, et al. Automatic detection of cognitive impairments through acoustic analysis of speech. Current Alzheimer Research. 2020;17(1):60–68. doi:10.2174/1567205017666200213094513.

108. König A, Linz N, Zeghari R, Klinge X, Tröger J, Alexandersson J, et al. Detecting Apathy in Older Adults with Cognitive Disorders Using Automatic Speech Analysis. Journal of Alzheimer's disease: JAD. 2019;69(4):1183–1193. doi:10.3233/JAD-181033.

109. König A, Linz N, Tröger J, Wolters M, Alexandersson J, Robert P. Fully Automatic Speech-Based Analysis of the Semantic Verbal Fluency Task. Dementia and Geriatric Cognitive Disorders. 2018;45(3-4):198–209. doi:10.1159/000487852.

110. Themistocleous C, Eckerström M, Kokkinakis D. Identification of Mild Cognitive Impairment From Speech in Swedish Using Deep Sequential Neural Networks. Frontiers in Neurology. 2018;9. doi:10.3389/fneur.2018.00975.

111. Wang T, Hong Y, Wang Q, Su R, Ng ML, Xu J, et al. Identification of Mild Cognitive Impairment among Chinese Based on Multiple Spoken Tasks. Journal of Alzheimer's Disease. 2021;82(1):185–204. doi:10.3233/jad-201387.

112. Konig A, Satt A, Sorin A, Hoory R, Derreumaux A, David R, et al. Use of Speech Analyses within a Mobile Application for the Assessment of Cognitive Impairment in Elderly People. Current Alzheimer Research. 2018;15(2):120–129. doi:10.2174/1567205014666170829111942.

113. Bertini F, Allevi D, Lutero G, Montesi D, Calzà L. Automatic Speech Classifier for Mild Cognitive Impairment and Early Dementia. ACM Transactions on Computing for Healthcare. 2022;3(1):1–11. doi:10.1145/3469089.

114. Verde L, De Pietro G, Sannino G. Artificial Intelligence Techniques for the Non-invasive Detection of COVID-19 Through the Analysis of Voice Signals. Arabian Journal for Science and Engineering. 2021;doi:10.1007/s13369-021-06041-4.

115. Verde L, De Pietro G, Ghoneim A, Alrashoud M, Al-Mutib KN, Sannino G. Exploring the Use of Artificial Intelligence Techniques to Detect the Presence of Coronavirus Covid-19 Through Speech and Voice Analysis. IEEE access : practical innovations, open solutions. 2021;9:65750–65757. doi:10.1109/ACCESS.2021.3075571.

116. Sharma G, Umapathy K, Krishnan S. Audio texture analysis of COVID-19 cough, breath, and speech sounds. Biomedical Signal Processing and Control. 2022;76. doi:10.1016/j.bspc.2022.103703.

117. Dar JA, Srivastava KK, Ahmed Lone S. Design and development of hybrid optimization enabled deep learning model for COVID-19 detection with comparative analysis with DCNN, BIAT-GRU, XGBoost. Computers in Biology and Medicine. 2022;150:106123. doi:10.1016/j.compbiomed.2022.106123.

118. Dang T, Han J, Xia T, Spathis D, Bondareva E, Siegele-Brown C, et al. Exploring Longitudinal Cough, Breath, and Voice Data for COVID-19 Progression Prediction via Sequential Deep Learning: Model Development and Validation. Journal of Medical Internet Research. 2022;24(6):e37004. doi:10.2196/37004.

119. Dash TK, Chakraborty C, Mahapatra S, Panda G. Gradient Boosting Machine and Efficient Combination of Features for Speech-Based Detection of COVID-19. IEEE Journal of Biomedical and Health Informatics. 2022;26(11):5364–5371. doi:10.1109/JBHI.2022.3197910.

120. Albadr MAA, Tiun S, Ayob M, Al-Dhief FT. Particle Swarm Optimization-Based Extreme Learning Machine for COVID-19 Detection. Cognitive Computation. 2022; p. 1–16. doi:10.1007/s12559-022-10063-x.

121. Rong P. A novel hierarchical framework for measuring the complexity and irregularity of multimodal speech signals and its application in the assessment of speech impairment in amyotrophic lateral sclerosis. Journal of Speech, Language, and Hearing Research. 2021;64(8):2996–3014. doi:10.1044/2021/JSLHR/20/00743.

122. Wang J, Kothalkar PV, Kim M, Bandini A, Cao B, Yunusova Y, et al. Automatic prediction of intelligible speaking rate for individuals with ALS from speech acoustic and articulatory samples. International Journal of Speech-Language Pathology. 2018;20(6):669–679. doi:10.1080/17549507.2018.1508499.

123. Tena A, Claria F, Solsona F, Meister E, Povedano M. Detection of Bulbar Involvement in Patients With Amyotrophic Lateral Sclerosis by Machine Learning Voice Analysis: Diagnostic Decision Support Development Study. JMIR medical informatics. 2021;9(3):e21331. doi:10.2196/21331.

124. Stegmann GM, Hahn S, Duncan CJ, Rutkove SB, Liss J, Shefner JM, et al. Estimation of forced vital capacity using speech acoustics in patients with ALS. Amyotrophic Lateral Sclerosis & Frontotemporal Degeneration. 2021;22(sup1):14–21. doi:10.1080/21678421.2020.1866013.

125. Rejaibi E, Komaty A, Meriaudeau F, Agrebi S, Othmani A. MFCC-based Recurrent Neural Network for automatic clinical depression recognition and assessment from speech. Biomedical Signal Processing and Control. 2022;71. doi:10.1016/j.bspc.2021.103107.

126. Lin Y, Liyanage BN, Sun Y, Lu T, Zhu Z, Liao Y, et al. A deep learning-based model for detecting depression in senior population. Frontiers in Psychiatry. 2022;13:1016676. doi:10.3389/fpsyt.2022.1016676.

127. Othmani A, Zeghina AO, Muzammel M. A Model of Normality Inspired Deep Learning Framework for Depression Relapse Prediction Using Audiovisual Data. Computer Methods and Programs in Biomedicine. 2022;226:107132. doi:10.1016/j.cmpb.2022.107132.

128. Hashim NNWN, Basri NA, Ezzi MAEA, Hashim NMHN. Comparison of classifiers using robust features for depression detection on Bahasa Malaysia speech. IAES International Journal of Artificial Intelligence. 2022;11(1):238–253. doi:10.11591/ijai.v11.i1.pp238-253.

129. Bourouhou A, Jilbab A, Nacir C, Hammouch A. Classification of Cardiovascular disease using dysphonia measurement in speech. Diagnostyka. 2021;22(1):31–38. doi:10.29354/diag/132586.

130. Pană MA, Busnatu SS, Serbanoiu LI, Vasilescu E, Popescu N, Andrei C, et al. Reducing the heart failure burden in romania by predicting congestive heart failure using artificial intelligence: proof of concept. Applied Sciences (Switzerland). 2021;11(24). doi:10.3390/app112411728.

131. Rao MV A, Yamini BK, Ketan J, Preetie Shetty A, Pal PK, Shivashankar N, et al. Automatic Classification of Healthy Subjects and Patients With Essential Vocal Tremor Using Probabilistic Source-Filter Model Based Noise Robust Pitch Estimation. Journal of Voice. 2021;doi:10.1016/j.jvoice.2021.01.009.

132. Suppa A, Asci F, Saggio G, Di Leo P, Zarezadeh Z, Ferrazzano G, et al. Voice Analysis with Machine Learning: One Step Closer to an Objective Diagnosis of Essential Tremor. Movement Disorders: Official Journal of the Movement Disorder Society. 2021;36(6):1401–1410. doi:10.1002/mds.28508.

133. Rozenstoks K, Novotny M, Horakova D, Rusz J. Automated Assessment of Oral Diadochokinesis in Multiple Sclerosis Using a Neural Network Approach: Effect

of Different Syllable Repetition Paradigms. IEEE transactions on neural systems and rehabilitation engineering: a publication of the IEEE Engineering in Medicine and Biology Society. 2020;28(1):32–41. doi:10.1109/TNSRE.2019.2943064.

134. Svoboda E, Bořil T, Rusz J, Tykalová T, Horáková D, Guttmann CRG, et al. Assessing clinical utility of machine learning and artificial intelligence approaches to analyze speech recordings in multiple sclerosis: A pilot study. Computers in Biology and Medicine. 2022;148:105853. doi:10.1016/j.compbiomed.2022.105853.

135. Ye W, Jiang Z, Li Q, Liu Y, Mou Z. A hybrid model for pathological voice recognition of post-stroke dysarthria by using 1DCNN and double-LSTM networks. Applied Acoustics. 2022;197:108934. doi:10.1016/j.apacoust.2022.108934.

136. Cai T, Ni H, Yu M, Huang X, Wong K, Volpi J, et al. DeepStroke: An efficient stroke screening framework for emergency rooms with multimodal adversarial deep learning. Medical Image Analysis. 2022;80:102522. doi:10.1016/j.media.2022.102522.

137. Chi NA, Washington P, Kline A, Husic A, Hou C, He C, et al. Classifying Autism From Crowdsourced Semistructured Speech Recordings: Machine Learning Model Comparison Study. JMIR pediatrics and parenting. 2022;5(2):e35406. doi:10.2196/35406.

138. Gao X, Ma K, Yang H, Wang K, Fu B, Zhu Y, et al. A rapid, non-invasive method for fatigue detection based on voice information. Frontiers in Cell and Developmental Biology. 2022;10:994001. doi:10.3389/fcell.2022.994001.

139. Al-Hameed S, Benaissa M, Christensen H, Mirheidari B, Blackburn D, Reuber M. A new diagnostic approach for the identification of patients with neurodegenerative cognitive complaints. PloS One. 2019;14(5):e0217388. doi:10.1371/journal.pone.0217388.

140. Roldan-Vasco S, Orozco-Duque A, Suarez-Escudero JC, Orozco-Arroyave JR. Machine learning based analysis of speech dimensions in functional oropharyngeal dysphagia. Computer Methods and Programs in Biomedicine. 2021;208:106248. doi:10.1016/j.cmpb.2021.106248.

141. Ditthapron A, Lammert AC, Agu EO. Continuous TBI Monitoring From Spontaneous Speech Using Parametrized Sinc Filters and a Cascading GRU. IEEE Journal of Biomedical and Health Informatics. 2022;26(7):3517–3528. doi:10.1109/JBHI.2022.3158840.

142. Alam MZ, Simonetti A, Brillantino R, Tayler N, Grainge C, Siribaddana P, et al. Predicting Pulmonary Function From the Analysis of Voice: A Machine Learning Approach. Frontiers in Digital Health. 2022;4. doi:10.3389/fdgth.2022.750226.

143. Farrús M, Codina-Filbà J, Reixach E, Andrés E, Sans M, Garcia N, et al. Speech-based support system to supervise chronic obstructive pulmonary disease patient status. Applied Sciences (Switzerland). 2021;11(17). doi:10.3390/app11177999.

144. Daqrouq K, Al-Qawasmi AR, Balamesh A, Alghamdi AS, Al-Amoudi MA. The Use of Arabic Vowels to Model the Pathological Effect of Influenza Disease by Wavelets. Computational and Mathematical Methods in Medicine. 2019;2019:1–8. doi:10.1155/2019/4198462.

145. Benba A, Jilbab A, Hammouch A. Voice assessments for detecting patients with neurological diseases using PCA and NPCA. International Journal of Speech Technology. 2017;20(3):673–683. doi:10.1007/s10772-017-9438-9.
